# Comparative analysis of mitochondrial genomes of two alpine medicinal plants of *Gentiana* (Gentianaceae)

**DOI:** 10.1371/journal.pone.0281134

**Published:** 2023-01-26

**Authors:** Kelsang Gyab Ala, Zhili Zhao, Lianghong Ni, Zhengtao Wang

**Affiliations:** 1 School of Pharmacy, Shanghai University of Traditional Chinese Medicine, Shanghai, China; 2 Mentseekhang, Traditional Tibetan Hospital, Lhasa, Tibet, China; 3 Institute of Chinese Materia Medica, Shanghai University of Traditional Chinese Medicine, Shanghai, China; Youngstown State University, UNITED STATES

## Abstract

*Gentiana crassicaulis* and *G*. *straminea* are alpine plants of *Gentiana* with important medicinal value and complex genetic backgrounds. In this study, the mitochondrial genomes (mtDNAs) of these two species were sequenced. The mtDNAs of *G*. *crassicaulis* and *G*. *straminea* are 368,808 and 410,086 bp long, respectively, 52 and 49 unique genes are annotated in the two species, and the gene arrangement varies widely. Compared to *G*. *crassicaulis*, *G*. *straminea* loses three effective genes, namely *atp6*, *trnG-GCC* and *trnV-GAC*. As a pseudogene, the *atp6* gene of *G*. *straminea* is incomplete, which is rare in higher plants. We detected 1696 and 1858 pairs of long repeats and 213 SSRs and 250 SSs in the mtDNAs of *G*. *crassicaulis* and *G*. *straminea*, respectively. There are 392 SNPs and 18 InDels between the two genomes, and syntenic sequence and structural variation analysis show low collinearity between the two genomes. Chloroplast DNA transferring to mtDNA is observed in both species, and 46,511 and 55,043 bp transferred segments containing three tRNA genes are identified, respectively. Comparative analysis of mtDNAs of *G*. *crassicaulis*, *G*. *straminea* and four species of Gentianales determined 18 core genes, and there is no specific gene in *G*. *crassicaulis* and *G*. *straminea*. The phylogenetic tree based on mtDNAs places Gentianaceae in a branch of Gentianales. This study is the first to analyze the mtDNAs of Gentianaceae, which could provide information for analysis of the structure of mtDNAs of higher plants and phylogenetic research of Gentianaceae and Gentianales.

## Introduction

The mitochondrion is an important organelle in eukaryotic cells and is intimately associated with energy generation and synthesis of fatty acids and some active proteins [1]. This genetically semi-autonomous organelle can encode genes related to its function [2]. Mitochondrial genomes (mtDNAs) of plants originated from independent living prokaryotes, and phylogenetic analysis found that they were closely related to Proteobacteria [3]. The mtDNA of *Arabidopsis thaliana* was first sequenced in 1997 [4]. With the development of sequencing technology, more and more plants have the mtDNA sequenced. The mtDNAs of plants mainly have the following characteristics [5]: (1) The genomes are large and genome size varies greatly among species. For example, the mtDNA of *Viscum scurruloideum* is only 66 kb in size [6], whereas *Larix sibirica* has the largest mtDNA size, with 11.7 Mb [7]. (2) Most mitochondria of plants encode roughly the same genes, but the order of genes varies greatly in higher plants [8, 9]. (3) Exchange of genes occurs between mtDNA and other genomes. The plant chloroplast genomes (cpDNAs) and mtDNAs are known to have extensive and widespread homologies due to sequence transfer [10, 11], and the transfer of cpDNA to mtDNA has been going on for at least 300 million years [12]. In addition, many mitochondrial genes have been found in the nuclear genomes of flowering plants [13]. (4) Genome fragments have been repeated and lost many times. (5) Recombination between repeated sequences complicates the structure of the genome. (6) The genome changes rapidly in structure and slowly in DNA sequences. Although the mtDNA evolves more slowly than nuclear genomes and cpDNAs and is generally used in studies of high classification levels, such as genera, families or even higher [14], it has abundant length polymorphism and unique advantages, which have been increasingly applied in molecular identification and systematic research of plants. With further research, the significance of the mtDNAs of plants is expected to be further developed.

*Gentiana* L. is the largest genus of Gentianaceae, widely distributed in temperate alpine regions of Europe, Asia and North America. The Qinghai–Tibet Plateau in southwest China is the distribution center of this genus and the region with the most abundant species diversity and endemic species of this genus [15]. *Gentiana crassicaulis* and *G*. *straminea* belong to *Gentiana* sect. *Cruciata*, have complex genetic backgrounds and are endemic to the Sino–Himalayan subregion [16]. The roots and flowers of *G*. *crassicaulis* and *G*. *straminea* are the sources of traditional Chinese medicine and Tibetan medicine used to treat rheumatism pain, stroke hemiplegia, muscle cramps, joint pain and damp-heat jaundice [17–19]. The cpDNA of *Gentiana* sect. *Cruciata* was studied [20–22], and the *rps16* pseudogene in the nonparasitic plants of the Asterids clade was identified for the first time. Based on sequences of chloroplast and mitochondrial fragments, a series of studies on molecular identification and systematic analysis were carried out [23, 24], and mtDNA showed significance in the identification of species in *Gentiana* sect. *Cruciata* [25]. At present, there is no report on the mtDNA of Gentianaceae.

In this study, we sequenced the mtDNAs of two Gentianaceae species, namely *G*. *crassicaulis* and *G*. *straminea*, for the first time, in order to provide data and information for the study of genome structure and species evolution of higher plants, and systematic analysis and molecular identification of Gentianaceae and Gentianales.

## Materials and methods

### Plant materials and mtDNA extraction

*Gentiana crassicaulis* and *G*. *straminea* were collected from Tibet, China, with GPS locations of E 97°14.003’, N 31°09.172’ and E 97°14.021’, N 31°09.174’, respectively. Also, the location of the specimens is not within any protected area. The plant samples were collected and transported to the laboratory in fresh condition. The roots of plants were frozen in liquid nitrogen and stored at –80 °C. The mtDNA was isolated from roots with a modified CTAB method [26].

### Genome sequencing and assembly

Genomic DNA was purified and fragmented to 300~500 bp to construct short-insert libraries, then sequenced on Illumina NovaSeq 6000 platform. Purified DNA was processed into 15-20k fragments and sequenced on PacBio Sequel II platform (LingEn Biotechnology Co., Ltd., Shanghai, China). Quality control of raw sequencing data was performed. To process the Illumina data, we performed the following steps: 1) removed adapter sequences in reads; 2) removed the bases containing non-AGCT at the 5’ end; 3) clipped the ends of reads with low quality (quality value <Q20); 4) removed reads containing up to 10% of N; 5) discarded small fragments with length <75 bp after adapter removal. PacBio data were processed as follows: 1) polymerase reads <200 bp were filtered out; 2) polymerase reads with quality <0.80 were filtered out; 3) subreads from the polymerase reads were extracted, and adapter sequences were filtered out; 4) subreads <200 bp were filtered out. Reads for each species-specific library have been deposited in the BioProject PRJNA831324.

Genome assembly involved the following steps: 1) Illumina sequencing data were assembled using GetOrganelle v1.7.1. BWA v0.7.17 was used to compare sequences of Illumina assembly to PacBio data. Then, the extracted PacBio data and Illumina data were mixed and assembled using SPAdes v3.14.1 [27]. The sequences with sufficiently high coverage depth and long assembly length were selected as candidate sequences, and the mitochondrial scaffold sequences were confirmed by comparison with the NT library, with sequences connected according to overlaps. 2) Clean reads were compared with the mtDNA sequence, and Pilon v1.23 was used to correct the bases.

### Genome annotation

A homologous comparison was carried out to predict the genes of genomes. The protein sequences of the NCBI mitochondrial reference genome were compared to genomes of the samples using BLAST+ 2.7.1 (e-value ≤ 1E-5). Poor alignment results were filtered out, and redundancy was removed. Then, the integrity of genes and the boundaries between exons and introns were manually corrected to obtain a high accuracy conservative gene set. The databases used to annotate coding genes included NR, Swiss-Prot, eggNOG, KEGG and GO. The tRNA and rRNA genes were predicted by tRNAscan-SE [28] and RNAmmer 1.2 [29], respectively. OrganellarGenomeDRAW (OGDRAW) was used to map the genomes [30].

### Repeat sequence analysis

There are a large number of repeats in mtDNAs of plants, with repeats of more than 30 bases considered as long repeats. REPuter [31] was used to analyse long repeats, and the parameters were set as follows: minimal repeat size, 30 bp; hamming distance, 3; maximum computed repeats, 5000. Four types of long repeats were identified as follows: F (Forward), R (Reverse), C (Complement) and P (Palindromic).

Simple sequence repeats (SSRs) exist widely in the mtDNAs of plants and are generally repeats composed of repeat units of 1–6 bp. MISA [32] was used to identify SSRs, in which the minimum repeats of mono-, di-, tri-, tetra-, penta- and hexanucleotides were set as 8, 5, 4, 3, 3 and 3, respectively. The minimum distance between two SSRs was set to 100 bp. Primer3 [33] was used to design primers for SSRs.

### Comparative analysis of mtDNAs of *G*. *crassicaulis* and *G*. *straminea*

MUMmer [34] was used to conduct a global comparison of the sequences of *G*. *crassicaulis* and *G*. *straminea*, find out the sites with differences between the sequences and conduct preliminary filtering to detect potential single nucleotide polymorphisms (SNPs). The 100 bp sequences on both sides of each SNP were extracted and compared with the assembly results using BLAT to verify the SNPs. SNPs <101 bp and those located in repeats were removed to obtain reliable SNPs.

LASTZ was used to compare the sequences of *G*. *crassicaulis* and *G*. *straminea*. The comparison data were processed by axt-correction, axtSort and axtBest programs to select the best results and obtain the initial Insertion/Deletions (InDels). Sequences of 150 bp on both sides of each InDel were compared with sequencing reads from another sample for verification, and reliable InDels were obtained by filtration.

MUMmer was used for comparing the mtDNAs of *G*. *crassicaulis* and *G*. *straminea* to confirm the large range of collinearity. LASTZ was then used to compare the regions to confirm the alignment and find areas of translocation (Trans), inversion (Inv) and translocation+inversion (Trans+Inv).

### Transfer of cpDNA to mtDNA

The cpDNA sequences of *G*. *crassicaulis* and *G*. *straminea* in our previous study [21] were downloaded from GenBank with IDs KY595458 and KJ657732, respectively. BLASTn (NCBI-BLAST-2.2.30+) was used to determine the homologous regions of mtDNA and cpDNA, with parameters set as consistency >90% and length >200 bp.

### Comparison of mtDNAs of Gentianales

Collinear and structural variation analyses were performed on the mtDNAs of *G*. *crassicaulis*, *G*. *straminea* and four species in Gentianales (*Asclepias syriaca*, *Cynanchum auriculatum*, *Rhazya stricta* and *Scyphiphora hydrophyllacea*) using MUMmer. Homologous genes in all species were identified as core genes (common genes). After removing core genes, dispensable genes (non-common genes) were obtained. Specific genes were genes specific only to a particular species. All dispensable genes were combined with core genes to form pan genes.

### Phylogenetic analysis

Phylogenetic relationships were analyzed based on the concatenated dataset of 29 protein-coding genes (*nad1*, *nad2*, *nad3*, *nad4*, *nad4L*, *nad5*, *nad6*, *nad7*, *nad9*, *cob*, *cox1*, *cox2*, *cox3*, *atp1*, *atp4*, *atp8*, *atp9*, *ccmB*, *ccmC*, *ccmFC*, *ccmFn*, *matR*, *rpl5*, *rpl16*, *rps1*, *rps3*, *rps4*, *rps12* and *rps13*) of 33 species, including *G*. *crassicaulis*, *G*. *straminea*, 30 species of other Asterids plants and 1 species of the outgroup (*Cycas taitungensis*). PhyML 3.0 was used to construct the phylogenetic tree by the maximum-likelihood method and Bayesian correction, with bootstrap calculations repeated 1000 times [35]. The GenBank IDs of mtDNAs used to construct the phylogenetic tree are shown in S1 Table.

## Results

### Genome sequencing and assembly

Illumina NovaSeq 6000 platform and PacBio Sequel II platform were used to sequence the mtDNAs of *G*. *crassicaulis* and *G*. *straminea*. For the Illumina sequencing, 4603.1 Mb and 4818.6 Mb raw data, 4327.2 Mb and 4578.3 Mb clean data were obtained from *G*. *crassicaulis* and *G*. *straminea*, respectively. The Q20 values for *G*. *crassicaulis* and *G*. *straminea* were 97.54% and 97.48, and the Q30 values for *G*. *crassicaulis* and *G*. *straminea* were 92.82% and 92.74%, respectively. The Illumina sequencing was paired-end and the insert size was 450 bp in both species sequencing. In regard to the PacBio sequencing, subreads with N50 = 9697 bp and subreads with N50 = 10079 bp were generated from *G*. *crassicaulis* and *G*. *straminea*, respectively. The information of raw data and quality control is shown in S2 Table. The mtDNAs of *G*. *crassicaulis* and *G*. *straminea* are 368,808 and 410,086 bp, respectively, which are the first two mtDNAs of Gentianaceae. Compared with other mtDNAs of Gentianales, they are smaller than those of *Asclepias syriaca* (682 kb) and *R*. *stricta* (548 kb) in Apocynaceae but similar to those of *Cynanchum auriculatum* (426 kb) in Asclepiadaceae and *Scyphiphora hydrophyllacea* (354 kb) in Rubiaceae. The GC contents of the two genomes are 44.83% and 44.55%, respectively, which are close to most higher plants [36, 37]. The mtDNA sequences of *G*. *crassicaulis* and *G*. *straminea* have been uploaded to GenBank with IDs OM320814 and OM328072, respectively.

### Genome annotation

**Fig 1:**
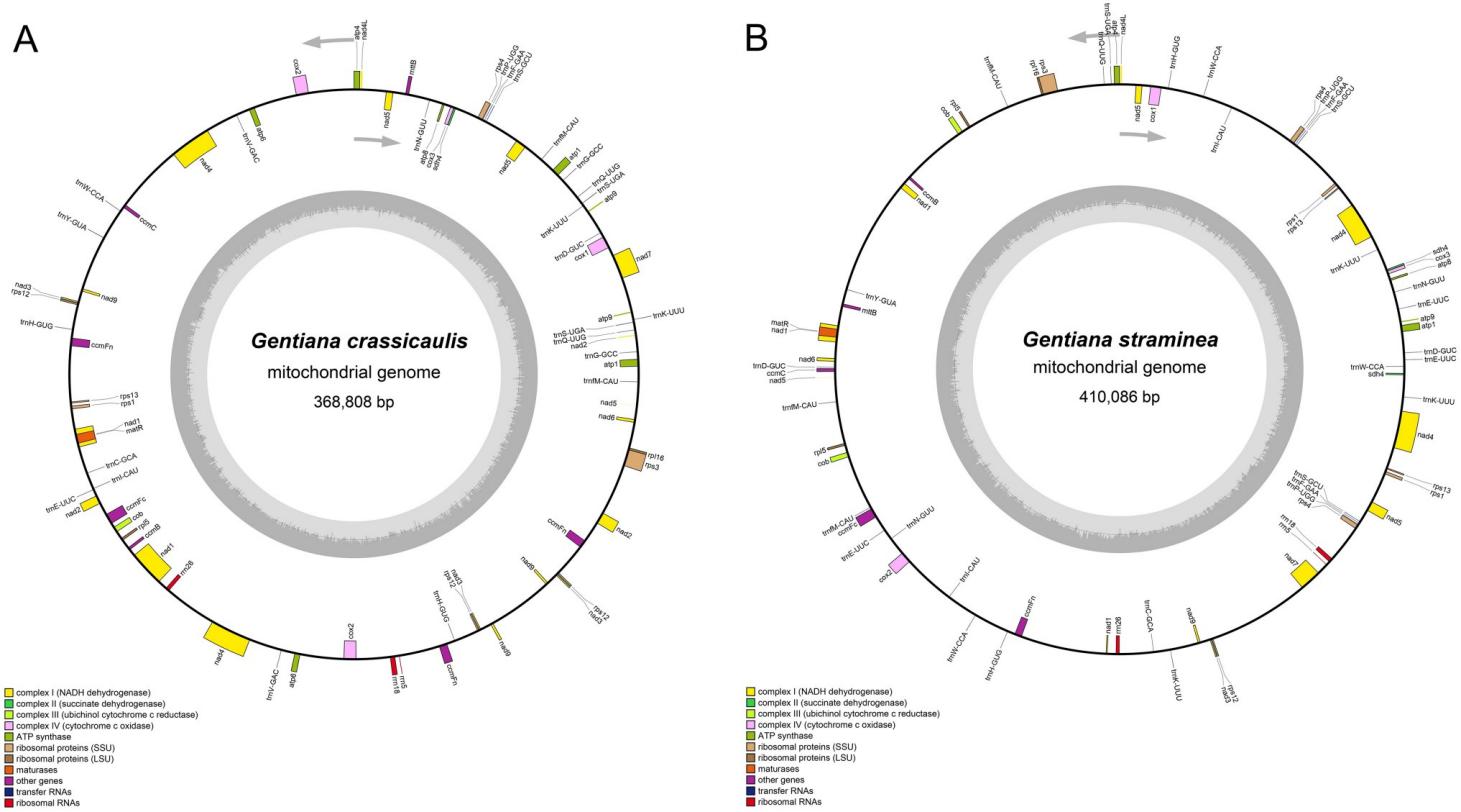
Mitochondrial genome map of *Gentiana crassicaulis* (A) and *G*. *straminea* (B). Genes (exons are shown as closed boxes) shown outside the curve are transcribed counterclockwise, whereas those inside are transcribed clockwise. Genes from the same protein complex are colored the same.

**Fig 2:**
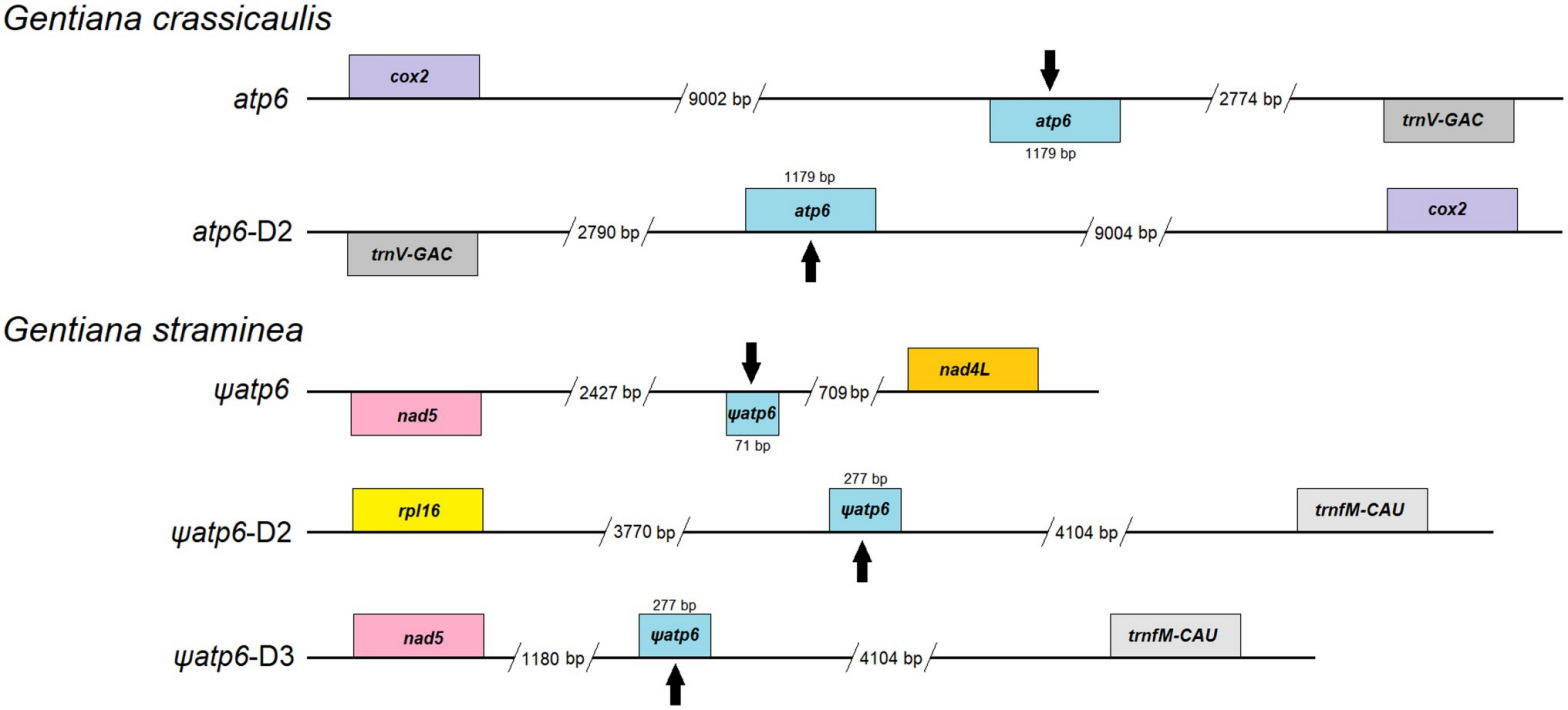
Location of *atp6* in mitochondrial genomes of *Gentiana crassicaulis* and *G*. *straminea*. This figure is not to scale.

Fifty-two unique genes were annotated in the mtDNA of *G*. *crassicaulis*. There are 32 protein-coding genes, 17 tRNA genes and 3 rRNA genes among these unique genes. In addition, there are two copies of five protein-coding genes, three copies of four protein-coding genes, and two copies of seven tRNA genes. Forty-nine unique genes were annotated in the mtDNA of *G*. *straminea*. There are 31 protein-coding genes, 15 tRNA genes and 3 rRNA genes among these unique genes. In addition, there are two copies of seven protein-coding genes, two copies of seven tRNA genes, and three copies of four tRNA genes (Table 1). The mtDNAs of *G*. *crassicaulis* and *G*. *straminea* are assembled into two single-ring molecules, and the order of the genes varies greatly (Fig 1). *Gentiana crassicaulis* contains three more unique genes than *G*. *straminea*, which are *atp6* (two copies), *trnG-GCC* (two copies) and *trnV-GAC* (two copies). The *atp6* gene of *G*. *straminea*, as a pseudogene, is incomplete and exists in the form of a fragment. There are three copies of *atp6* with the length of 71, 277 and 277 bp, respectively (Fig 2). Primers were designed for PCR amplification of *atp6* regions in *G*. *straminea*, which further verified the accuracy of the sequences (S1 Data). An incomplete pseudogene of *atp6*, which can be regarded as missing, is rare in mtDNAs of angiosperms.

**Table 1:** Gene component of the mitochondrial genomes of *Gentiana crassicaulis* and *G*. *straminea*.

ID	*G*. *crassicaulis*	*G*. *straminea*
Complex I (NADH dehydrogenase)	*nad1*, *nad2*, *nad3*(×3), *nad4*(×2), *nad4L*, *nad5*, *nad6*, *nad7*, *nad9*(×3)	*nad1*, *nad2*, *nad3*, *nad4*(×2), *nad4L*, *nad5*, *nad6*, *nad7*, *nad9*
Complex II (Succinate dehydrogenase)	*sdh4*	*sdh4*(×2)
Complex III (Ubiquinol cytochrome *c* reductase)	*cob*	*cob*(×2)
Complex IV (Cytochrome *c* oxidase)	*cox1*, *cox2*(×2), *cox3*	*cox1*, *cox2*, *cox3*
ATP synthase	*atp1*(×2), *atp4*, *atp6*(×2), *atp8*, *atp9*(×2)	*atp1*, *atp4*, *atp8*, *atp9*
Ribosomal proteins (SSU)	*rps1*, *rps3*, *rps4*, *rps12*(×3), *rps13*	*rps1*(×2), *rps3*, *rps4*(×2), *rps12*, *rps13*(×2)
Ribosomal proteins (LSU)	*rpl5*, *rpl16*	*rpl5*(×2), *rpl16*
Maturases	*matR*	*matR*
Other genes	*ccmB*, *ccmC*, *ccmFc*, *ccmFn*(×3), *mttB*	*ccmB*, *ccmC*, *ccmFc*, *ccmFn*, *mttB*
Transfer RNAs	*trnC-GCA*, *trnD-GUC*, *trnE-UUC*, *trnF-GAA*, *trnfM-CAU*(×2), *trnG-GCC*(×2), *trnH-GUG*(×2), *trnI-CAU*, *trnK-UUU*(×2), *trnN-GUU*, *trnP-UGG*, *trnQ-UUG*(2), *trnS-GCU*, *trnS-UGA*(×2), *trnV-GAC*(×2), *trnW-CCA*, *trnY-GUA*	*trnC-GCA*, *trnD-GUC*(×2), *trnE-UUC*(×3), *trnF-GAA*(×2), *trnfM-CAU*(×3), *trnH-GUG*(×2), *trnI-CAU*(×2), *trnK-UUU*(×3), *trnN-GUU*(×2), *trnP-UGG*(×2), *trnQ-UUG*, *trnS-GCU*(×2), *trnS-UGA*, *trnW-CCA*(×3), *trnY-GUA*
Ribosomal RNAs	*rrn5*, *rrn18*, *rrn26*	*rrn5*, *rrn18*, *rrn26*
Genome size (bp)	368,808	410,086
GC content (%)	44.83	44.55
Gene number*	45; 24; 3	38; 30; 3
Gene total length (bp)	42,699	35,796

* The numbers represent the number of protein-coding genes, tRNA genes and rRNA genes, respectively.

There are nine genes with introns in the mtDNAs of the two species, respectively, namely *nad1*, *nad2*, *nad4*, *nad5*, *nad7*, *cox1*, *cox2*, *ccmFc* and *rps3*. The number of introns is the same, and most introns are the same or similar in length (S3 Table). It is worth noting that *nad2* and *nad5* of *G*. *crassicaulis* contain two *trans*-splicing introns each, and *nad1*, *nad2* and *nad5* of *G*. *straminea* contain one, three and two *trans*-splicing introns, respectively. The structures of these NADH dehydrogenase genes containing *trans*-splicing introns vary greatly in different plants and first appeared in primitive vascular plants, which may be molecular markers of plant evolution [38–40].

### Repeat sequence analysis

**Fig 3:**
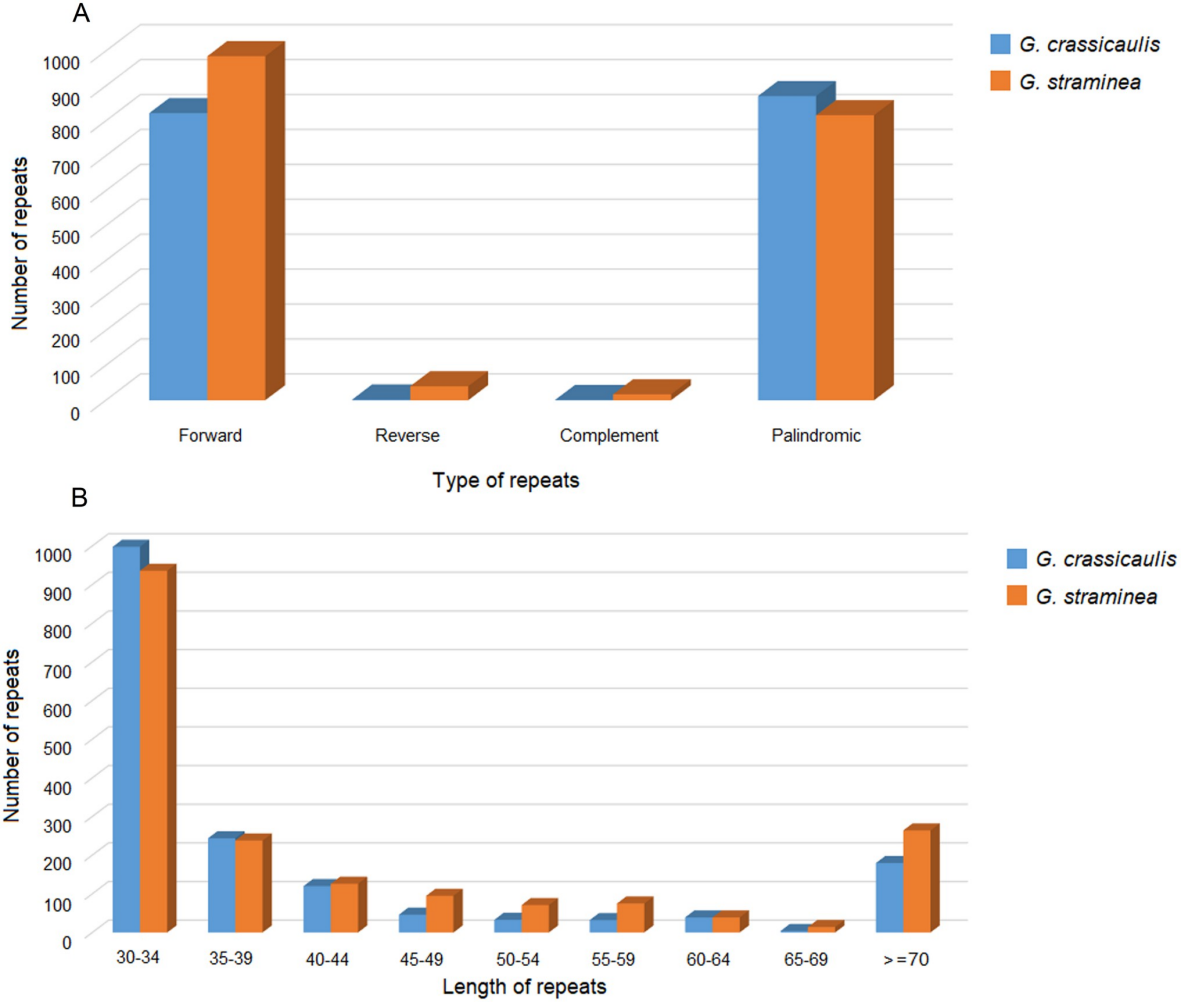
Large repeat sequence characteristics of mitochondrial genomes of *Gentiana crassicaulis* and *G*. *straminea*.

We detected 1696 pairs of long repeats in the mtDNA of *G*. *crassicaulis*, including 822 pairs of forward repeats, 2 pairs of reverse repeats, 1 pair of complement repeats, and 871 pairs of palindromic repeats. Lengths of repeat units range from 30 to 9683 bp. In addition, we detected 1858 pairs of long repeats in the mtDNA of *G*. *straminea*, including 985 pairs of forward repeats, 40 pairs of reverse repeats, 17 pairs of complement repeats, and 816 pairs of palindromic repeats. Lengths of repeat units range from 30 to 30,663 bp. In the two genomes, the number of repeats with a length of 30 to 34 bp is the largest, as 998 (58.5%) and 936 (50.4%), respectively (Fig 3, S4 Table).

As an important component of mtDNA, repeated sequences contribute much of the genome structure complexities through mediation of homologous recombinations [41]. Generally, large repeats (>1000 bp) with high sequence similarity tend to recombine more frequently, medium repeats (100–1000 bp) recombine occasionally, and small repeats (<100 bp) rarely [42]. The active repeat-mediated intragenomic recombinations in *G*. *crassicaulis* and *G*. *straminea* were detected with long reads database following Dong et al. [43]. The repeated sequences in *G*. *crassicaulis* and *G*. *straminea* mtDNA contain small proportions (6.9% and 9.7%, respectively) of large and medium repeats, and no evidence of recombination activity was detected other than the 13 and 16 repeats shown in S4 Table. The length of the repeats and recombination rate are correlated, with no recombination evidence detected for repeat sequences of shorter than 100 bp. All the repeats with recombination activity showed low recombination frequencies less than 12%. The low recombination level provides evidence for the predominant master conformation in both species. Such low recombination frequency has also been seen in other plant mtDNAs, for example, in *Silene conica* and *Silene noctiflora* [44], tens of large repeats induced recombinations at a frequency around 5%; in *Nymphaea colorata* [43], ten recombinationally active repeat pairs were detected from 886,982 repeat pairs examined; and in *Vigna radiata* [45], no recombination activity was detected.

**Fig 4:**
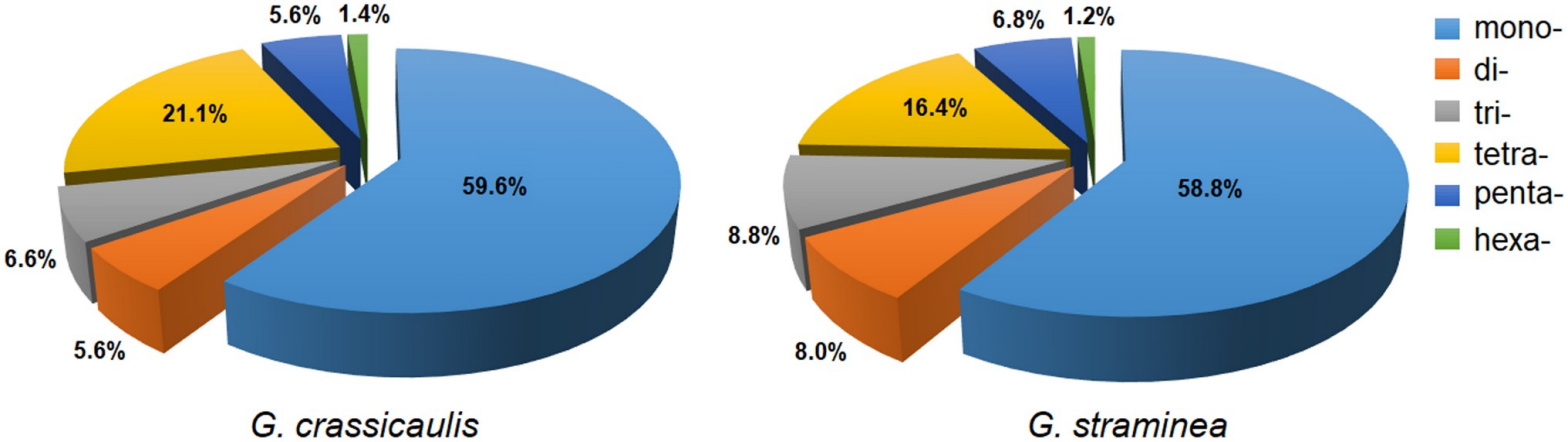
SSR characteristics of mitochondrial genomes of *Gentiana crassicaulis* and *G*. *straminea*.

SSR is a common molecular marker to evaluate the genetic diversity of species. A total of 213 SSRs were detected in the mtDNA of *G*. *crassicaulis*, including 127 mononucleotide SSRs (59.6%), 12 dinucleotide SSRs (5.6%), 14 trinucleotide SSRs (6.6%), 45 tetranucleotide SSRs (21.1%), 12 pentanucleotide SSRs (5.6%) and 3 hexanucleotide SSRs (1.4%). A total of 250 SSRs were detected in the mtDNA of *G*. *straminea*, including 147 mononucleotide SSRs (58.8%), 20 dinucleotide SSRs (8.0%), 22 trinucleotide SSRs (8.8%), 41 tetranucleotide SSRs (16.4%), 17 pentanucleotide SSRs (6.8%) and 3 hexanucleotide (1.2%) (Fig 4). The number of A/T mononucleotide SSRs is the largest, with 118 and 134, respectively. Among all SSRs, only one SSR is longer than 20 bp (Table 2). The total SSRs of *G*. *crassicaulis* and *G*. *straminea* are 3361 and 3700 bp long, respectively, and primers have been designed for SSRs (S5 Table).

**Table 2:** SSRs identified in the mitochondrial genomes of *Gentiana crassicaulis* and *G*. *straminea*.

Type		*G*. *crassicaulis*		*G*. *straminea*	
		num	num (≥20 bp)	num	num (≥20 bp)
Mono-	A/T	118	0	134	0
	C/G	9	0	13	0
Di-	AC/GT	1	0	1	0
	AG/CT	2	0	2	0
	AT/AT	9	0	17	1
Tetra-	AAG/CTT	3	0	6	0
	AAT/ATT	5	0	7	0
	ACT/AGT	4	0	5	0
	AGC/CTG	2	0	2	0
	AGG/CCT	0	0	1	0
	ATC/ATG	0	0	1	0
Tetra-		45	0	41	0
Penta-		12	0	17	0
Hexa-		3	0	3	1

### Comparative analysis of mtDNAs of *G*. *crassicaulis* and *G*. *straminea*

Comprehensive comparisons were performed in terms of synteny and homologous sequences of the two *Gentiana* species. SNP refers to the polymorphism of DNA sequences caused by a single nucleotide variation. InDel refers to the insertion and deletion of small fragments of the genome. A comparison of the mtDNAs of *G*. *crassicaulis* and *G*. *straminea* reveals 392 SNPs, including 354 SNPs in intergenic regions and 38 SNPs in coding regions, including 15 synonymous mutations, 22 non-synonymous mutations and 1 premature stop codon mutation. There are no synonymous or non-synonymous mutations in the start and stop codons of the SNPs in the coding region (S6 Table). In addition, there are 18 InDels, all of which are in intergenic regions and do not cause gene mutations (S7 Table).

**Fig 5:**
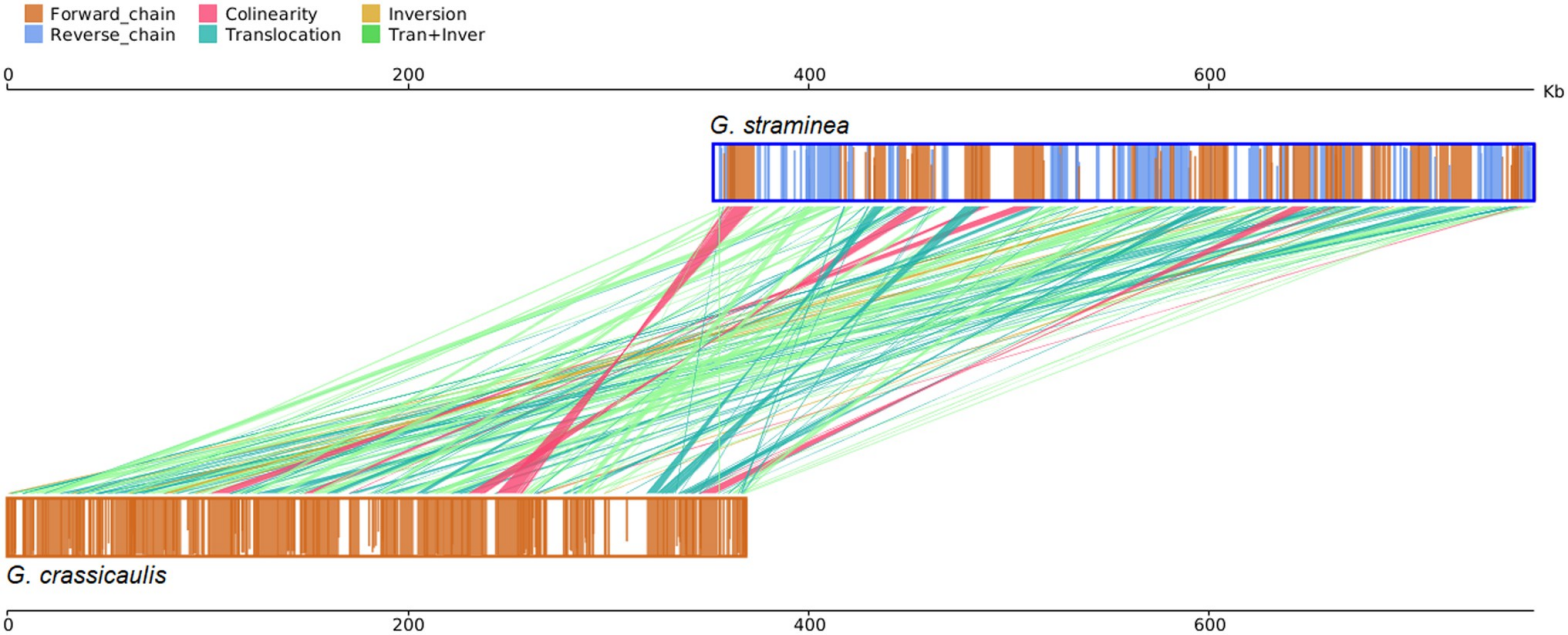
Collinear relationship between the mitochondrial genomes of *Gentiana crassicaulis* and *G*. *straminea*. The upper and lower bars represented the mitochondrial genomes. Dark-orange and blue regions in each bar represented the forward and reverse direction of the aligned genome, respectively. White regions in each bar represent the sequences that could not be aligned to the other genome. Lines between the two bars indicated the syntenic types and locations: magenta, blue-green, dark yellow and light-green represent collinearity, translocation, inversion and translocation+inversion, respectively.

**Fig 6:**
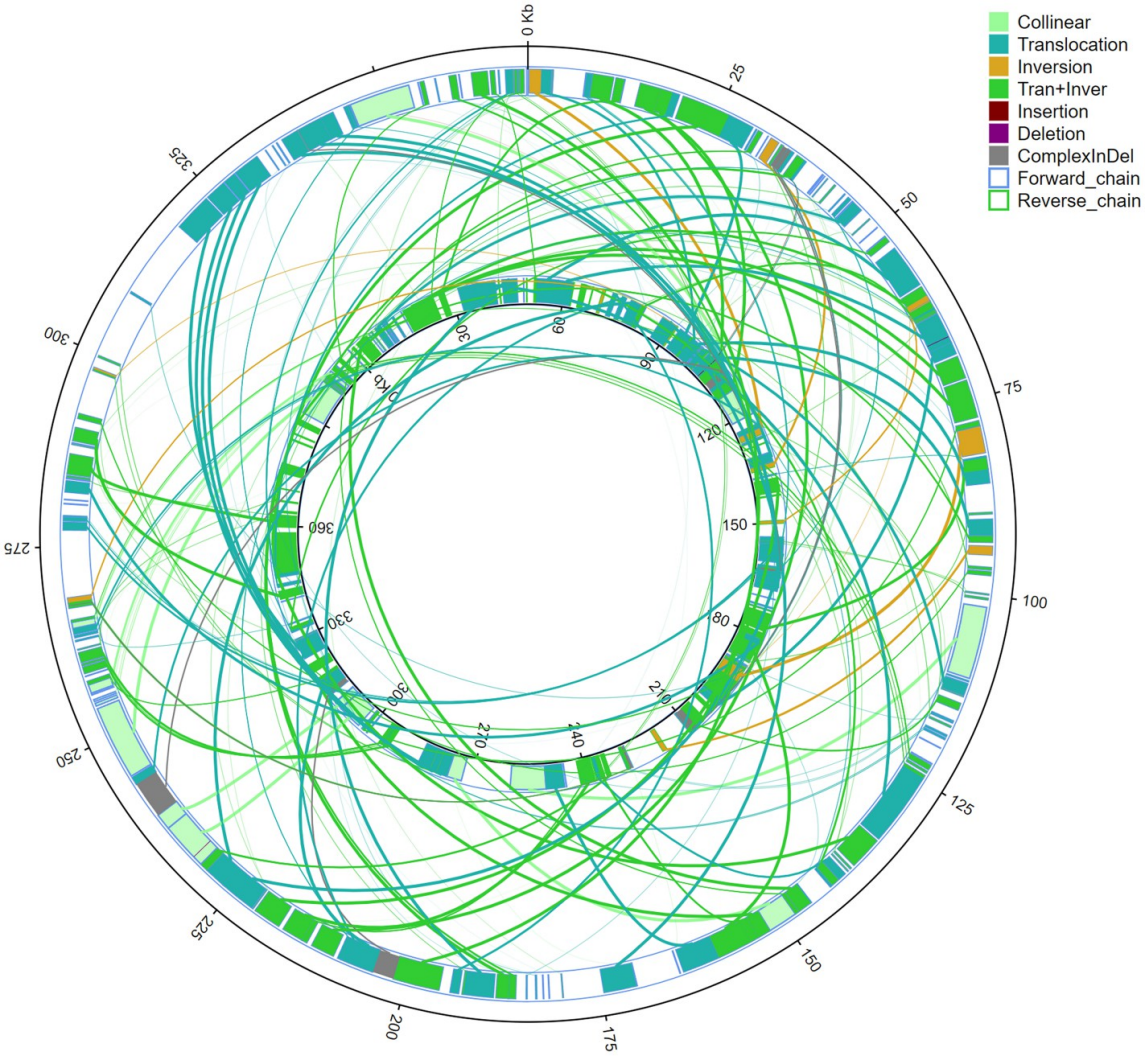
Structural variation map of mitochondrial genomes of *Gentiana crassicaulis* and *G*. *straminea*. The inner circle is the *G*. *crassicaulis* genome, and the outer circle is the *G*. *straminea* genome. Collinear: homologous region; translocation: translocation region; inversion: inverted region; tran+inver: translocation and inversion region; insertion: insertion region with length ≥50 bp; deletion: deletion region with length ≥50 bp; complexInDel: region without alignment but corresponding to location; forward_chain: forward chain of a genomic sequence, at which time the gene sitz markers increase clockwise; reverse_chain: the reverse chain of a genomic sequence, where the gene coordinates increase counterclockwise.

By comparing homologous sequences between genomes, insertion and deletion could be observed, and the structural variation of genomes in the process of evolution. The coverages of collinear comparison of mtDNAs of *G*. *crassicaulis* and *G*. *straminea* are 60.89% and 54.81%, respectively (S8 Table). The number of comparison regions is 209, and the numbers of collinearity, Trans, Inv and Trans+Inv are 14, 87, 8 and 100, respectively (Fig 5, S9 Table). Structural variation study further clarifies the differences between the two genomes, and there are lots of Trans, Inv, Trans+Inv and Indels (Fig 6, S10 Table). The results show significant structural differences between the mtDNAs of *G*. *crassicaulis* and *G*. *straminea*, resulting in a distinct difference in gene order (Fig 1). And despite gene content being conserved, the numbers of copies changed, especially in some tRNAs (Table 1). Structural differences in mtDNA from the same genus of plants are relatively common, for example, a conventional single molecular structure was resolved in *Broussonetia papyrifera* (Moraceae) and *B*. *monoica* while a double-ring structure was resolved in *B*. *kaempferi* with gene content showing consistency except for a few tRNA genes [46]. As a whole, homologous regions across the two *Gentiana* mtDNAs revealed high compositional and low structural conservation. According to Fu et al. 2021 [47], the time of divergence of *G*. *straminea* and its relative species in *Gentiana* sect. *Cruciata* was about 4.14 million years ago. The rearrangement rate of mtDNAs of the two species in this study was estimated using the number of rearrangement events (Trans / Inv -related events events) by dividing it by two times the divergence time and a rate of ~19.9 rearrangement events per million years was obtained, which is not surprising given prior studies of angiosperms [48]. The mtDNAs of seed plants show high rates of rearrangement of at least 600-fold and rearrangement rates may vary greatly even within a single genus [48, 49].

### Transfer of cpDNA to mtDNA

**Fig 7:**
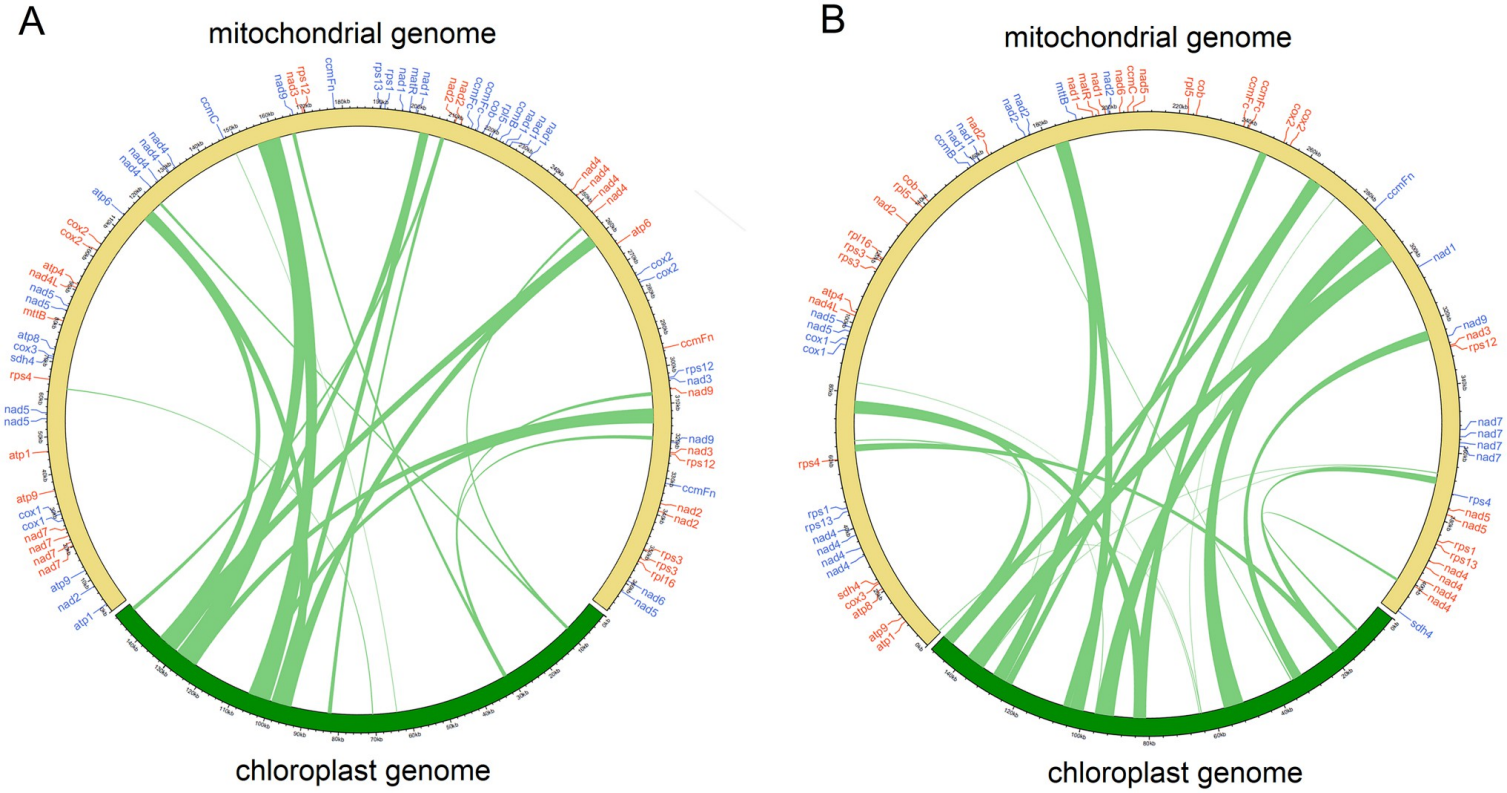
Gene exchange in chloroplast genome and mitochondrial genome of *Gentiana crassicaulis* (A) and *G*. *straminea* (B). The green lines within the circle show the regions of the chloroplast genome those have been inserted into different locations of the mitochondrial genome.

Recent research has shown information exchange and transmission between chloroplasts and mitochondria in plants [50]. The transfer of cpDNA to mitochondria occurs mostly in the ancestors of vascular plants [43]. The length of mtDNA of *G*. *crassicaulis* is 2.48 times that of its cpDNA (148,757 bp), and the length of mtDNA of *G*. *straminea* is 2.75 times that of its cpDNA (148,991 bp). Both species show transfer of cpDNA to mtDNA (Fig 7). In the cpDNA and mtDNA of the two species, 21 and 24 transferred segments with total lengths of 46,511 and 55,043 bp are identified with a similarity greater than 90%, respectively. The length of the transferred segments in *G*. *crassicaulis* is 141–6551 bp and the length of the transferred segments in *G*. *straminea* is 109–6176 bp. Some of these transferred segments of cpDNA carry genes, in which the protein-coding genes usually become non-functional, while the tRNA genes mostly remain functional. This phenomenon has also been found in *Magnolia biondii* [42], *Asclepias syriaca* [50], *R*. *stricta* [51], *Salvia miltiorrhiza* [52] and other plants. Three tRNA genes, including *trnW-CCA*, *trnV-GAC* and *trnI-CAU*, from the cpDNA were identified in the mtDNA of *G*. *crassicaulis*. Three tRNA genes from the cpDNA, namely *trnW-CCA*, *trnI-CAU* and *trnN-GUU*, were also found in the mtDNA of *G*. *straminea* (S11 Table). Intracellular gene transfer of DNA from the chloroplast to the mitochondrion in higher plants is a common phenomenon, and the transferred fragments differ greatly. In the analysis of the homologs in 79 species of chloroplast-derived mitochondrion fragments, fragment containing *trnW-CCA* was also observed in *Nelumbo nucifera*, *Nicotiana tabacum*, *Vitis vinifera* and *Liriodendron tulipifera*, while the most common fragments were those that contain *trnN-GTT*, *trnH-GTG* and *trnM-CAT* [53].

### Comparison of mtDNAs of Gentianales

**Fig 8:**
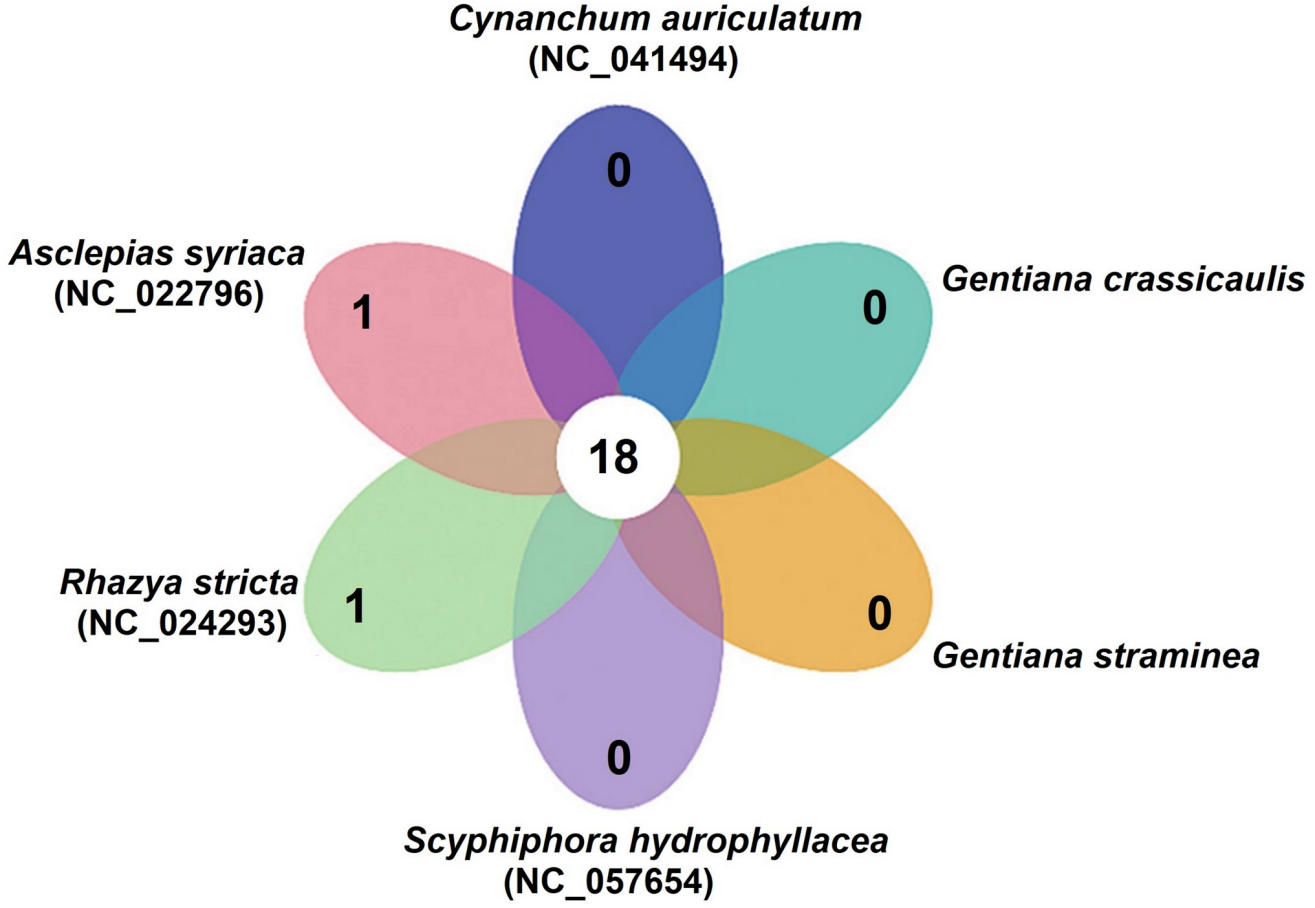
Core and specific genes of six Gentianales plants.

Collinearity and structural variation analysis were performed based on the mtDNAs of *G*. *crassicaulis*, *G*. *straminea* and four species in Gentianales, namely *Asclepias syriaca*, *R*. *stricta*, *Cynanchum auriculatum* and *Scyphiphora hydrophyllacea*. The coverages of collinear comparison only accounted for 18.40–26.02% of the genome of *G*. *crassicaulis* and 16.79–25.95% of the genome of *G*. *straminea*, respectively. A large number of Trans, Inv and Indels were found in pairwise comparison, and the proportion of collinearity in the comparison areas was only 12.14–14.58% for *G*. *crassicaulis* and 6.71–13.79% for *G*. *straminea*, respectively (S1 and S2 Figs). The results show that the mtDNAs of the two *Gentiana* species are markedly different from other species in Gentianales. There are 42 pan genes (all non-shared genes and shared genes) in the mtDNAs of these six species in Gentianales, including 18 core genes. *Asclepias syriaca* and *R*. *stricta* have one specific gene each (Fig 8, S12 Table). These core genes and specific genes could provide the basis for studying the functional differences among different species in Gentianales.

### Phylogenetic analysis

**Fig 9:**
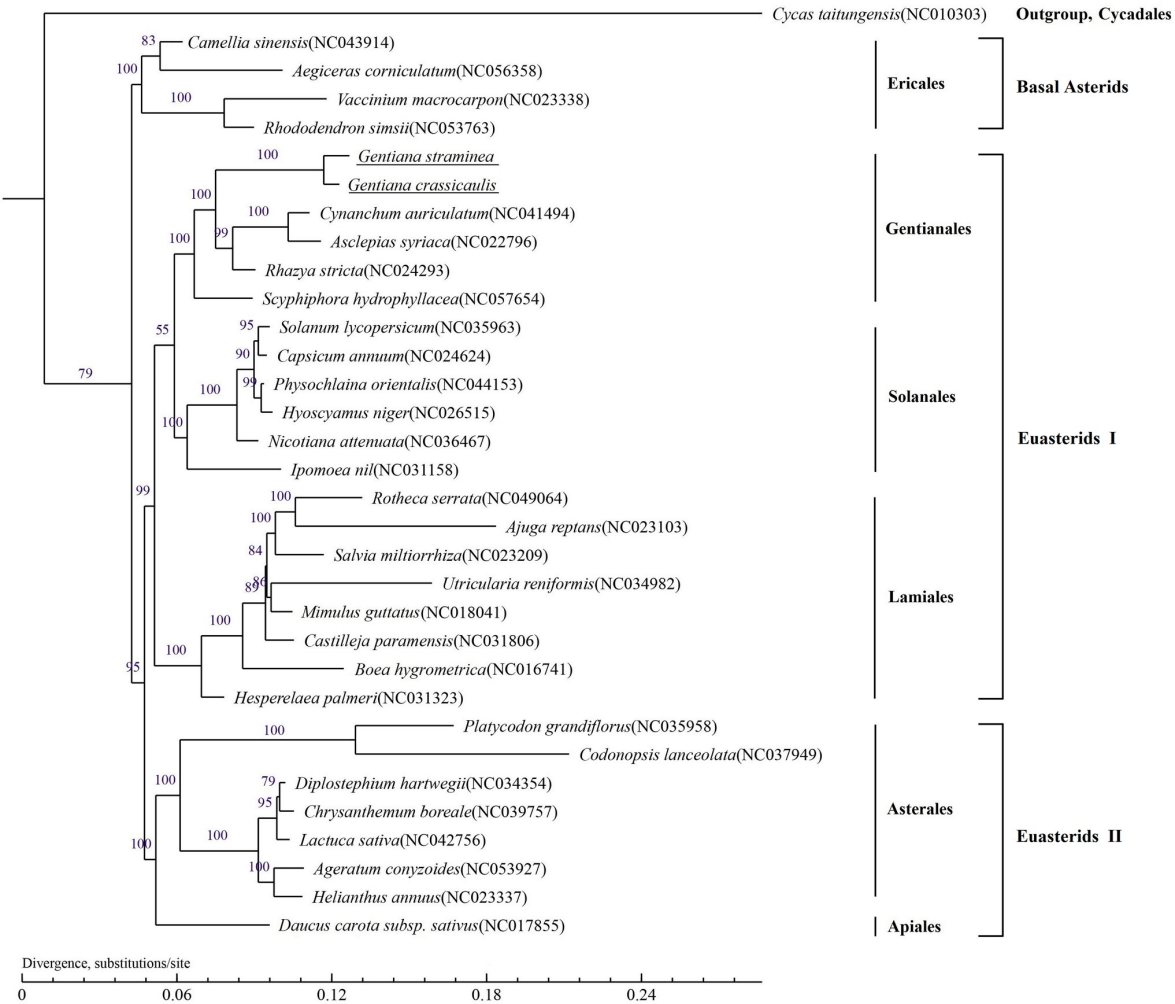
Molecular phylogenetic tree of 33 species based on the complete mitochondrial genomes. The tree was constructed via a maximum-likelihood analysis using PhyML 3.0 with 1000 bootstrap replications.

*Gentiana crassicaulis* and *G*. *straminea* belong to the Gentianaceae family in the Asterids clade. To identify its phylogenetic position, the mtDNAs of 32 species belonging to six orders of Asterids were selected for phylogenetic analysis, including Ericales, Gentianales, Solanales, Lamiales, Asterales and Apiales (S1 Table). *Cycas taitungensis*, from Cycadales, was set as an outgroup. In the phylogenetic tree, bootstrap values of 20 out of 31 nodes are 100%, and bootstrap values of 7 nodes are ≥90%. *Gentiana crassicaulis* and *G*. *straminea* are closely clustered on a branch with a bootstrap value of 100%. Gentianaceae is located in the branch of Gentianales, which is consistent with the classification of APG IV. The topologies of the phylogenetic tree mainly consist of two clades, one of which is composed of Euasterids I (Solanales, Gentianales and Lamiales) and Euasterids II (Asterales and Apiales), and the other is composed of basal Asterids (Ericales) (Fig 9). The systematic relationship of these orders is consistent with that based on cpDNAs in a previous study [20]. It should be noted that the relationship of Solanales, Gentianales and Lamiales within Euasterids I remains poorly resolved [54, 55]. In this study, the result supports the position of Gentianales as the sister of Solanales, but the node connecting the three orders has a relatively low bootstrap value of 73%. This problem also exists in cpDNA-based phylogenetic tree [20]. The system relationship among the orders in Euasterids I needs further analysis.

## Discussion

*Gentiana crassicaulis* and *G*. *straminea* are closely related species of *Gentiana* sect. *Cruciata* and their cpDNAs show a consistent structure and high sequence similarity [21], unlike their mtDNAs, which show great structural differences and are also very different from those of other species in Gentianales. Studies have shown that mtDNAs of plants differentiate structurally quickly, resulting in a large loss of collinear and shared DNA [56, 57]. For example, only 51% of the mtDNA of *Vigna radiata* is homologous to that of *Glycine max*, despite their differentiation for only 19 million years [58]. The sizes of mtDNAs of plants vary greatly among different families and genera [9] but also among closely related species. For example, *Gossypium arboreum*, woody cotton, and *Gossypium hirsutum*, upland cotton, differ in mtDNA size by 65.6 kb. Moreover, only about half of mtDNA sequences are identical among different populations of *Silene venosa* [44]. There is no positive correlation between genome size and gene number. The size of the mtDNA of *G*. *crassicaulis* is smaller than that of *G*. *straminea*, but it contains more genes than *G*. *straminea*. For another example, the mtDNA of *Welwitschia mirabilis* is more than twice as large as the mtDNA of *Cycas taitungensis*, but it has fewer genes than *Cycas taitungensis* [49]. The main reason is that the mtDNA is paradoxically composed of intergenic regions that are evolving more rapidly than the surrounding DNA, resulting in sparse gene arrangement and an increasingly large and complex genome [8].

In this study, the deletion of *atp6* is found in the mtDNA of *G*. *straminea*. There are widespread gene deletions in the mtDNAs of plants during evolution. The number of protein-coding genes decreases with plant evolution, and the lost genes are usually ribosome protein genes and succinate dehydrogenase genes [13]. For example, *sdh2* was lost in the earliest evolution of plants, and *rps9*, *rps11 and rps16* were lost in higher plants when they differentiated from lower plants. Subsequently, in the differentiation of monocotyledons and dicotyledons, monocotyledons lost *rps12*, *sdh3* and *sdh4*, and dicotyledons lost *rps2*. Furthermore, at differentiation of different classes and genera of higher plants, some genes may have been lost [8]. The respiratory chain complex V genes in the mtDNAs of plants include *atp1*, *atp4*, *atp6*, *atp8* and *atp9*. Deletion of these genes has also been detected in some plants. For example, *Chondrus crispus* loses *atp1* [59], *Arabidopsis thaliana* and *Nicotiana tabacum* lose *atp4* [4, 60], *Nicotiana tabacum* loses *atp8* [61], and *Citrullus lanatus* has one additional source of five edited sites in the form of a partial *atp9* pseudogene [62]. The *atp6* gene is absent in the single-cell green alga *Chlamydomonas reinhardtii* [4]. Moreover, some plants, such as *Lotus japonicus*, contain the *atp6* pseudogene in addition to the complete *atp6* gene [63]. There is no complete *atp6* gene in *G*. *straminea*, and only the *atp6* pseudogene exists, which is rare in angiosperms. The *atp6* gene encodes the largest F_0_ subunit (A subunit) in the F_1_F_0_-ATP synthase system [64]. Male sterility of rice, rape and radish is known to be related to *atp6* mutation [65–67]. In our previous study, the *rps16* pseudogene of the Asterids clade nonparasitic plant was first identified in the cpDNA of *G*. *straminea* [20]. Is there a correlation between the specificity of genomic structure and the environment of *G*. *straminea* as a cold-tolerant alpine plant? How does the loss of relevant genes affect its function? All of these questions need to be further studied.

Genetic information is exchanged between the genomes of plant organelles (mitochondria and chloroplasts). Chloroplasts are highly genetically conserved with few foreign DNA, but the transfer of cpDNA to mtDNA occurs frequently [68]. For example, the expansion of mtDNA of *Cucurbita pepo* may be related to the transfer of a large number of chloroplast sequences [62]. Seventeen chloroplast sequences ranging in size from 32 bp to 6.7 kb were detected in mtDNA of rice, and they were scattered throughout mtDNA, accounting for 6.3% of the total length of mtDNA. No common motifs or secondary structures were found in the boundary regions of the integration of these foreign DNA sequences. It is speculated that the integration of the chloroplast DNA sequences is not site-specific and may be the result of non-homologous or random recombination [69]. Meanwhile, the DNA sequences transferred from chloroplasts to mitochondria which are functional are limited to tRNA genes. Due to multiple sequence changes and the absence of RNA editing, transferred protein-coding sequences are often non-functional [9].

Loss of tRNA genes is also associated with the evolution of plants, which may occur during the differentiation of bryophytes and gymnosperms [63, 70]. There are huge differences in tRNA genes between the mtDNAs of higher plants and those of lower plants, and there are also differences in tRNA genes among plants of different families or genera of higher plants, as well as among the populations of the same species [44, 71]. An important feature of tRNA genes in the mtDNAs of plants is that they have dual origins; that is, some tRNA genes are directly inherited from mitochondrial ancestors, while the others are derived from the transferred sequences of chloroplasts. Therefore, tRNA genes in mtDNAs of plants could be divided into "tRNA originating from mitochondria" and "tRNA originating from chloroplasts" by source [72]. For example, in the mtDNA of *Boea hygrometrica*, half of the 28 tRNA genes were identified as originating from the cpDNA [40]. In the mtDNA of *Spirodela polyrhiza*, 4 out of 19 tRNA genes originated from the cpDNA [36]. In this study, three tRNA genes are derived from the cpDNA in the mtDNA of *G*. *crassicaulis* and *G*. *straminea*, respectively, and *G*. *crassicaulis* contains two more unique tRNA genes than *G*. *straminea*.

The genetic background of *Gentiana* sect. *Cruciata* is complex, and there are hybrid individuals of different species [73], which makes it difficult to identify related species of this section. Previous studies showed incomplete concerted evolution of ITS sequences in many species of *Gentiana* sect. *Cruciata*, such as *G*. *crassicaulis* [23, 24]. MtDNAs and cpDNAs are maternal-inherited with moderate sizes and no heterozygote, and rapid progress has been made in the application of DNA barcoding developed from them in the identification of traditional Chinese medicine [5, 74]. A previous study also found that *nad*1/b-c and *nad*5/d-e from mtDNAs have implications for species identification in *Gentiana* sect. *Cruciata* [25]. In our study, a large number of repeats and SSR markers are developed based on the mtDNAs of *G*. *crassicaulis* and *G*. *straminea*, which may be further used for molecular identification and genetic diversity of *Gentiana* and Gentianaceae.

## Conclusions

In this study, the mtDNAs of alpine plants *G*. *crassicaulis* and *G*. *straminea* of Gentianaceae are sequenced for the first time, and the sequences and structures are compared and analyzed.

The mtDNAs of *G*. *crassicaulis* and *G*. *straminea* are 368,808 and 410,086 bp long, respectively, 52 and 49 unique genes are annotated in the two species. And the pseudogene *atp6* was identified in *G*. *straminea*, which is rare in higher plants. A large number of repeats and SSR markers are developed and cpDNA transferring to mtDNA is observed in both species. The phylogenetic tree based on mtDNAs supports the traditional taxonomic view of Gentianaceae. The work could provide data for analysis of mtDNA structure and species evolution of higher plants and data for phylogenetic study and species identification of Gentianaceae.

## Supporting information

S1 Data Validation of sequences of *atp6* gene. (DOCX)

S1 Fig Collinear relationship between mitochondrial genomes of *Gentiana crassicaulis* (*G*. *straminea*) and other four Gentianales plants. (DOCX)

S2 Fig Structural variation map of mitochondrial genomes of *Gentiana crassicaulis* (*G*. *straminea*) and other four Gentianales plants. (DOCX)

S1 Table GenBank IDs of mitochondrial genomes used to construct the phylogenetic tree. (DOCX)

S2 Table Illumina NovaSeq 6000 sequencing results and PacBio Sequel II sequencing results. (DOCX)

S3 Table Characteristics of introns and exons in mitochondrial genes of *Gentiana crassicaulis* and *G*. *straminea*. (DOCX)

S4 Table Long repeats of mitochondrial genomes of *Gentiana crassicaulis* and *G*. *straminea*. (XLSX)

S5 Table SSRs of mitochondrial genomes of *Gentiana crassicaulis* and *G*. *straminea*. (XLSX)

S6 Table *Gentiana straminea* SNP annotation results of the reference sequence of *G*. *crassicaulis*. (DOCX)

S7 Table *Gentiana straminea* InDel annotation results of the reference sequence of *G*. *crassicaulis*. (DOCX)

S8 Table Coverage statistics of collinearity comparison of *Gentiana crassicaulis* (Target) and *G*. *straminea* (Query). (DOCX)

S9 Table Information of collinearity comparison of *Gentiana crassicaulis* (Target) and *G*. *straminea* (Query). (DOCX)

S10 Table Information of structural variation analysis of *Gentiana crassicaulis* (Target) and *G*. *straminea* (Query). (DOCX)

S11 Table Information exchange and transfer between chloroplasts and mitochondria in *Gentiana crassicaulis* and *G*. *straminea*. (DOCX)

S12 Table Pan genes of 6 Gentianales plants. (DOCX)
