# Supplementary material for: Comparative analysis of mitochondrial genomes of two alpine medicinal plants of Gentiana (Gentianaceae)
Source: PLoS One. 2023 Jan 26;18(1):e0281134. doi: 10.1371/journal.pone.0281134 (PMC9879513; doi:10.1371/journal.pone.0281134)
Supplement: S1 Data — (DOCX) [file pone.0281134.s001.docx]

**S1 Data** Validation of sequences of *atp6* gene.

***Gentiana crassicaulis***

**(1) 113198-114376（1179 bp） *atp6***

TTACTTCGGGAAGGAAAGAGGAGGCCTTTGCTCTATCTGCTTCTTCCTCTTACGAGTGGCTATCGCACCTCGCTTTTGTTCAATTATAAATAAATAAGCACCTTGATGGAGATTTGTAGCATCATTCAAGTAAATACAGATTAAGATCGTAGAAACATGAGCTTGTGATATAGCTACACCTAATTCCAGACCGGTTAATGCAAGAACTATAAATAAAGGACCAAGATCTCCTATGAAATAGAAAAGATCATTCATACATAGCATAGTCCAAGCGAACCCACTCAAAATCTTTACTGAACTATGACCGGCCATCATATTAGCAAATAAACGTATTCCTGAGCTTAATGCGCGAAAACAATGAGGGATTAGCTCAAGGAGTACTAAAAAGGGTGCTAACGGCAGTGGGACTCCTGCGGGTAATAAAAAGCTTAAAAAATGAAGCCCATTTCTTTGAAATCCCACTATAGTAATGCCAATAAAAATAGAAAATGAGAGACCTAAAGTAATGAGAAAATGACTTGTAACTGTGAAGCTATAAGGTATCATACCCTGGGGATTACGAAATAACAAAAAAGTAAAAGTGACCGAGATGCAAGGGGAAAACTTTTGTTTAACATTTCCGGAAAGACCACCTATTTGTTCGTTTACCGGGTTCAGCACGAAATCATAAATAAGCTCTACCAAGGATTGCCAAGCATTTGGTACTGAGTTTCCTCCTCCCTTTTTAGTAACAAAATAAAATAAAAGTAGGACCAAACTGAGAGTTAGCAGCATAAAGAAAGATGGATTTGTGAATGAGAAATACAAGTCTCCTAGTTTCATAGAAATCAATGGGAGAATGGTAAATTGGTCAAGTGGGCTGGGAATTTGAGGGACCTCTGGAAAGTTTAGCAAGAAGTTTTGATTAGCTACTCGTGGATCTACTAGCAGGGACTCAATATAAGGGATCTCGTCAGGTATACGTGCCTCGAAGTGCCTGTCATAGTAAAATTCTACACCGGGTGTCCGCGCTCCCGAACTACTGGGAGACTGCGGGGCAGCTGAGACTTCTACGCGACCAGTTCTTTCATAAATCTTAATAAGAGATTTGAACTTCCTTGAGACTTCCACGCGATCAGTAACTTTATAAAGCTCAATAATAGATCTGAACTTCCTTTCTAAAATCTTCCTTTTCTTCAT

*The sequence marked red is shared by *Gentiana straminea*.

**(2) 264276-265454（1179 bp） *atp6*-D2**

ATGAAGAAAAGGAAGATTTTAGAAAGGAAGTTCAGATCTATTATTGAGCTTTATAAAGTTACTGATCGCGTGGAAGTCTCAAGGAAGTTCAAATCTCTTATTAAGATTTATGAAAGAACTGGTCGCGTAGAAGTCTCAGCTGCCCCGCAGTCTCCCAGTAGTTCGGGAGCGCGGACACCCGGTGTAGAATTTTACTATGACAGGCACTTCGAGGCACGTATACCTGACGAGATCCCTTATATTGAGTCCCTGCTAGTAGATCCACGAGTAGCTAATCAAAACTTCTTGCTAAACTTTCCAGAGGTCCCTCAAATTCCCAGCCCACTTGACCAATTTACCATTCTCCCATTGATTTCTATGAAACTAGGAGACTTGTATTTCTCATTCACAAATCCATCTTTCTTTATGCTGCTAACTCTCAGTTTGGTCCTACTTTTATTTTATTTTGTTACTAAAAAGGGAGGAGGAAACTCAGTACCAAATGCTTGGCAATCCTTGGTAGAGCTTATTTATGATTTCGTGCTGAACCCGGTAAACGAACAAATAGGTGGTCTTTCCGGAAATGTTAAACAAAAGTTTTCCCCTTGCATCTCGGTCACTTTTACTTTTTTGTTATTTCGTAATCCCCAGGGTATGATACCTTATAGCTTCACAGTTACAAGTCATTTTCTCATTACTTTAGGTCTCTCATTTTCTATTTTTATTGGCATTACTATAGTGGGATTTCAAAGAAATGGGCTTCATTTTTTAAGCTTTTTATTACCCGCAGGAGTCCCACTGCCGTTAGCACCCTTTTTAGTACTCCTTGAGCTAATCCCTCATTGTTTTCGCGCATTAAGCTCAGGAATACGTTTATTTGCTAATATGATGGCCGGTCATAGTTCAGTAAAGATTTTGAGTGGGTTCGCTTGGACTATGCTATGTATGAATGATCTTTTCTATTTCATAGGAGATCTTGGTCCTTTATTTATAGTTCTTGCATTAACCGGTCTGGAATTAGGTGTAGCTATATCACAAGCTCATGTTTCTACGATCTTAATCTGTATTTACTTGAATGATGCTACAAATCTCCATCAAGGTGCTTATTTATTTATAATTGAACAAAAGCGAGGTGCGATAGCCACTCGTAAGAGGAAGAAGCAGATAGAGCAAAGGCCTCCTCTTTCCTTCCCGAAGTAA

*The sequence marked red is shared by *Gentiana straminea*.

***Gentiana straminea***

**(1) 101264-101334（71 bp） *ψatp6***

CCGCCCCCAAGCCAGTGTCCATCTTCATGAAATTACATTGGTAGACGGGTTGGGACCAGCCTTATTTCTATCCCGGTGAGCAATGTTCCTTAGGCTTAGTAGGGCTCGAACCTACAATATCACCGTTATGAGCGGTACGTTTCAACCAATTAAACTATAAGCCCCTACGAATCTCTACATGCAATTCGCTTACTTCGGGAAGGAAAGAGGAGGCCTTTGCTCTATCTGCTTCTTCCTCTTACGAGTGGCTATCGCACCTCTTACGAGCATTAAATCCTCCGTGGTCCGTTTCTTCATCTTCCGGTGTAGACAACTATAGGCCAAACCGCCTGGACACTAGGCCCAAGGATCGCTTAGCACCGTGGACCACAGACCACTAAATACATTTCGCGTAAGAGGTACGTAACCCACTCGATAGTCTAAAGAGCGGCGTAACCCACTCGACAGATAACCCTACGAGAGCAAAGAACTCGCTCGCAAGAATACTCGCTTCGCTCCGCTACAC

Primer 1：CCGCCCCCAAGCCAGTGT

primer 2：GTGTAGCGGAGCGAAGCGAGTATT

* The sequence marked in red is the sequence of *atp6.*

*The underlined parts are the regions of primers.

**(2) 124440-124716（277 bp）*ψatp6*-D2**

**(3) 208068-208344（277 bp）*ψatp6*-D3**

AATAGCCGGGGTTCGCAGACGTACTATCAGTACATATCCAAATAGGCAGCCTTCTAAAGTGAAAAGGCTCCTTTTCGATGTTCCTGAAATCCATTTGAATACTGGATTGGTAGAGGTAGGCCACTCAACCCTTACTTGAGAGATGCACTCATTGGCTGTTCTCGAAGAACCCGCTCGGGAGGAAGCATTCCACCTCCCGTGCTTTCCGTTGGTCAACAACCAACAACTCGTTTAGTTCTTGAAGAGTCTCTCTCTTTTTTTGGGGAGCAGAGCAGTCAAAGAATGAACCAAGGGAGAGTATGAAGAAAAGGAAGATTTTAGAAAGGAAGTTCAGATCTATTATTGAGCTTTATAAAGTTACTGATCGCGTGGAAGTCTCAAGGAAGTTCAAATCTCTTATTAAGATTTATGAAAGAACTGGTCGCGTAGAAGTCTCAGCTGCCCCGCAGTCTCCCAGTAGTTCGGGAGCGCGGACACCCGGTGTAGAATTTGACTATGACAGGCACTTCGAGGCACGTATACCTGACGAGATCCCTTATATTGAGTCCCTGCTAGTAGATCCACGAGTAGCTAATCTCTTGTATATGGCATCTAGAAAGAGTTATAATTATAGTTCTAGTAGATATTATTGTGATAAACCAAGTGAGCTTGAGAATGAGAAGAAGGATAAGGGAATGCAGACTCCATTGAGTAAGCGTGAAAGGGAT

Primer 1：AATAGCCGGGGTTCGCAGAC

primer 2：ATCCCTTTCACGCTTACTCAATGG

* The sequence marked in red is the sequence of *atp6.*

*The underlined parts are the regions of primers.
