# Supplementary material for: Comparative analysis of mitochondrial genomes of two alpine medicinal plants of Gentiana (Gentianaceae)
Source: PLoS One. 2023 Jan 26;18(1):e0281134. doi: 10.1371/journal.pone.0281134 (PMC9879513; doi:10.1371/journal.pone.0281134)
Supplement: S2 Fig — (DOCX) [file pone.0281134.s003.docx]

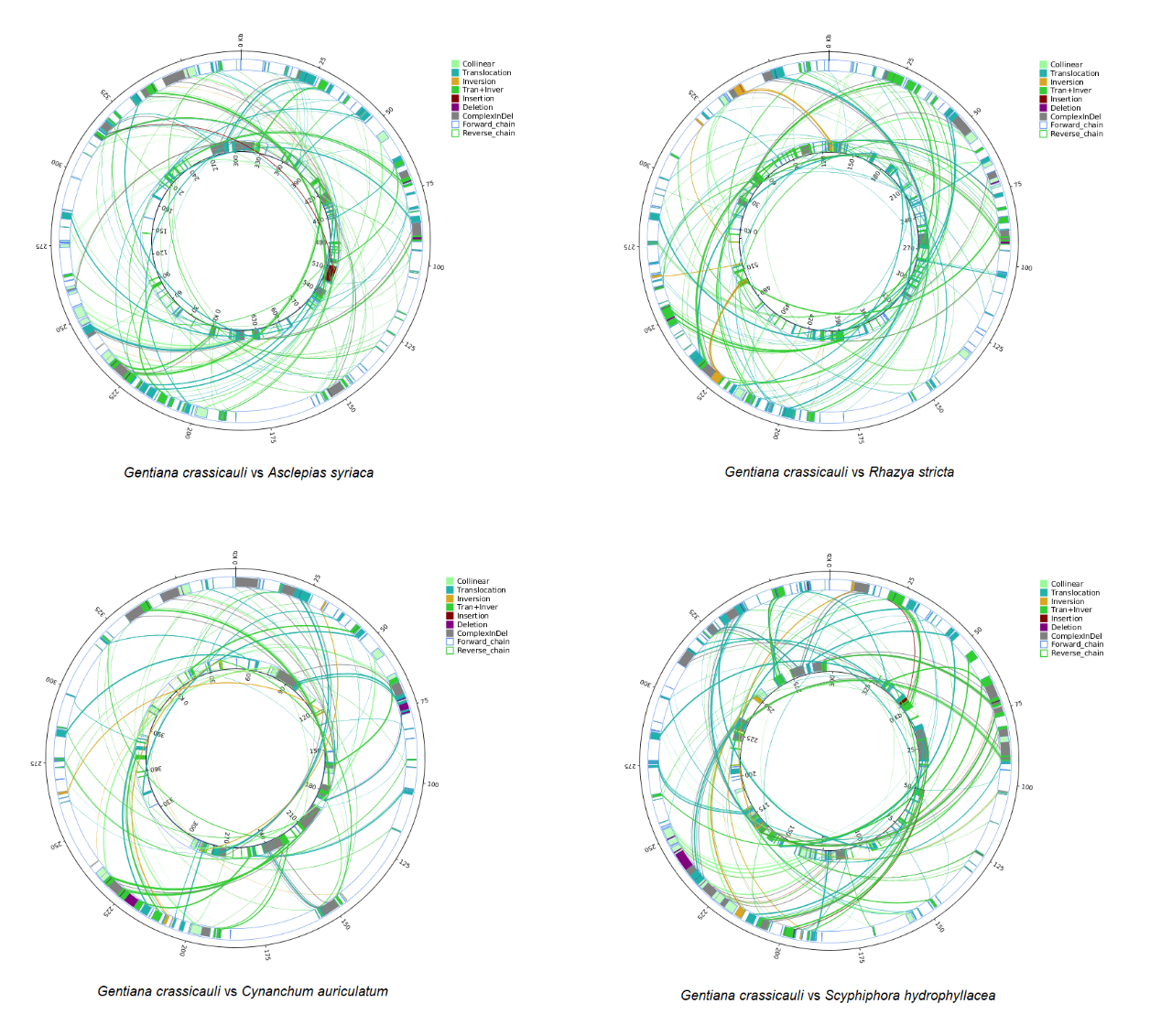


**S2-1** **Fig** Structural variation map of mitochondrial genomes of *G. crassicaulis* and other four Gentianales plants, respectively.


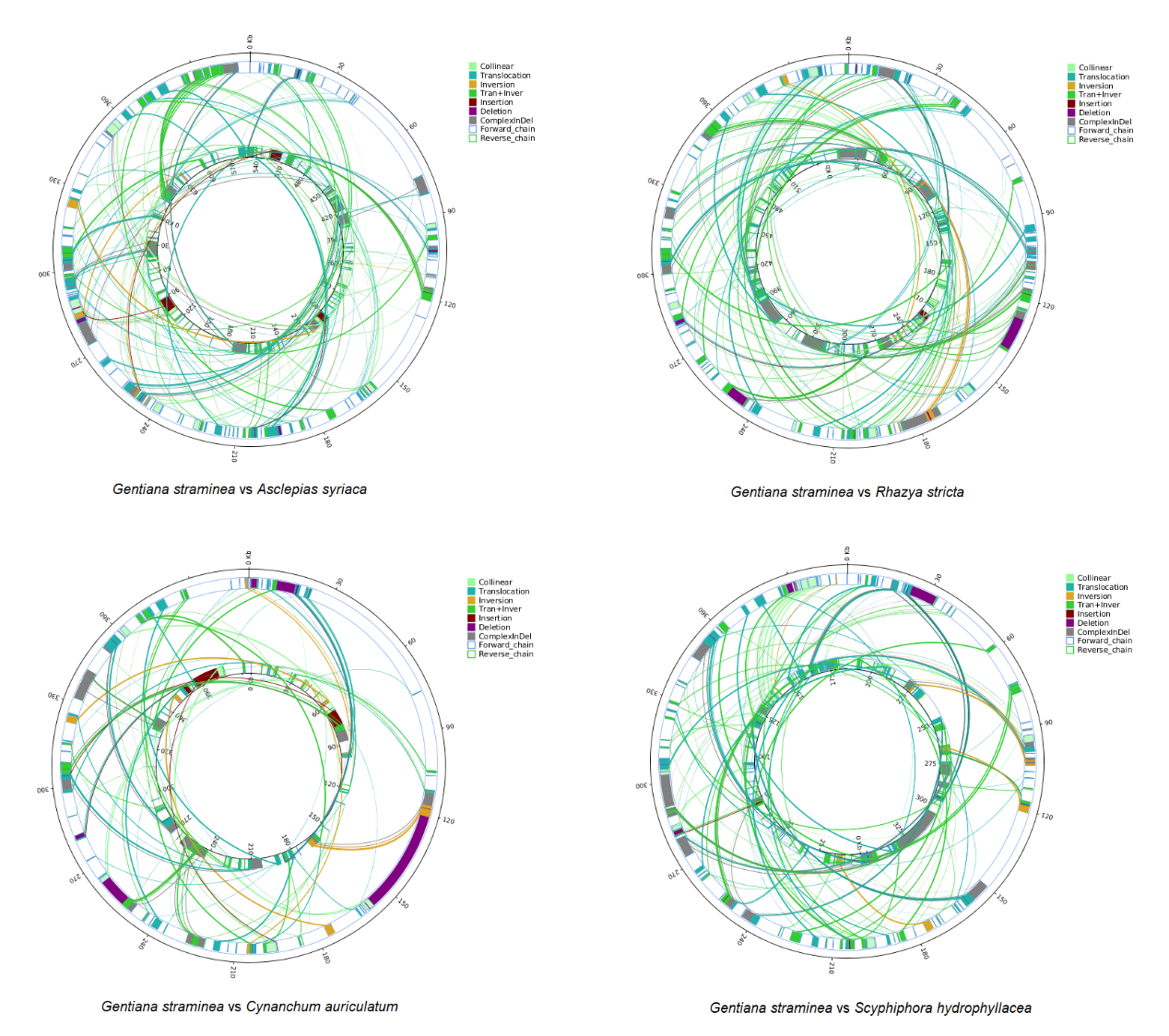


**S2-2 Fig** Structural variation map of mitochondrial genomes of *G. straminea* and other four Gentianales plants, respectively.
