# Supplementary material for: Comparative analysis of mitochondrial genomes of two alpine medicinal plants of Gentiana (Gentianaceae)
Source: PLoS One. 2023 Jan 26;18(1):e0281134. doi: 10.1371/journal.pone.0281134 (PMC9879513; doi:10.1371/journal.pone.0281134)
Supplement: S1 Table — (DOCX) [file pone.0281134.s004.docx]

**S1 Table** GenBank IDs of mitochondrial genomes used to construct the phylogenetic tree.

| **Species** | **GenBank ID** |
| --- | --- |
| [*Capsicum annuum*](https://www.ncbi.nlm.nih.gov/genome/10896?genome_assembly_id=227510) | [NC_024624.1](https://www.ncbi.nlm.nih.gov/nuccore/NC_024624.1) |
| [*Hyoscyamus niger*](https://www.ncbi.nlm.nih.gov/genome/37025?genome_assembly_id=229757) | [NC_026515.1](https://www.ncbi.nlm.nih.gov/nuccore/NC_026515.1) |
| [*Ipomoea nil*](https://www.ncbi.nlm.nih.gov/genome/46552?genome_assembly_id=293413) | [NC_031158.1](https://www.ncbi.nlm.nih.gov/nuccore/NC_031158.1) |
| [*Nicotiana attenuata*](https://www.ncbi.nlm.nih.gov/genome/13243?genome_assembly_id=336542) | [NC_036467.1](https://www.ncbi.nlm.nih.gov/nuccore/NC_036467.1) |
| [*Physochlaina orientalis*](https://www.ncbi.nlm.nih.gov/genome/83237?genome_assembly_id=649035) | [NC_044153.1](https://www.ncbi.nlm.nih.gov/nuccore/NC_044153.1) |
| [*Solanum lycopersicum*](https://www.ncbi.nlm.nih.gov/genome/7?genome_assembly_id=336537) | [NC_035963.1](https://www.ncbi.nlm.nih.gov/nuccore/NC_035963.1) |
| [*Asclepias syriaca*](https://www.ncbi.nlm.nih.gov/genome/11317?genome_assembly_id=248246) | [NC_022796.1](https://www.ncbi.nlm.nih.gov/nuccore/NC_022796.1) |
| [*Cynanchum auriculatum*](https://www.ncbi.nlm.nih.gov/genome/43866?genome_assembly_id=490626) | [NC_041494.1](https://www.ncbi.nlm.nih.gov/nuccore/NC_041494.1) |
| [*Rhazya stricta*](https://www.ncbi.nlm.nih.gov/genome/22340?genome_assembly_id=206121) | [NC_024293.1](https://www.ncbi.nlm.nih.gov/nuccore/NC_024293.1) |
| [*Scyphiphora hydrophyllacea*](https://www.ncbi.nlm.nih.gov/genome/92406?genome_assembly_id=913367) | [NC_057654.1](https://www.ncbi.nlm.nih.gov/nuccore/NC_057654.1) |
| *Gentiana crassicaulis* | OM320814 |
| *Gentiana straminea* | OM328072 |
| [*Ajuga reptans*](https://www.ncbi.nlm.nih.gov/genome/23970?genome_assembly_id=49393) | [NC_023103.1](https://www.ncbi.nlm.nih.gov/nuccore/NC_023103.1) |
| *Mimulus guttatus* | [NC_018041.1](https://www.ncbi.nlm.nih.gov/nuccore/NC_018041.1) |
| [*Castilleja paramensis*](https://www.ncbi.nlm.nih.gov/genome/50341?genome_assembly_id=292884) | [NC_031806.1](https://www.ncbi.nlm.nih.gov/nuccore/NC_031806.1) |
| [*Boea hygrometrica*](https://www.ncbi.nlm.nih.gov/nuccore/NC_016741.1) | [NC_016741.1](https://www.ncbi.nlm.nih.gov/nuccore/NC_016741.1) |
| [*Hesperelaea palmeri*](https://www.ncbi.nlm.nih.gov/genome/35376?genome_assembly_id=283966) | [NC_031323.1](https://www.ncbi.nlm.nih.gov/nuccore/NC_031323.1) |
| [*Rotheca serrata*](https://www.ncbi.nlm.nih.gov/genome/92482?genome_assembly_id=913443) | [NC_049064.1](https://www.ncbi.nlm.nih.gov/nuccore/NC_049064.1) |
| [*Salvia miltiorrhiza*](https://www.ncbi.nlm.nih.gov/genome/11235?genome_assembly_id=49477) | [NC_023209.1](https://www.ncbi.nlm.nih.gov/nuccore/NC_023209.1) |
| [*Utricularia reniformis*](https://www.ncbi.nlm.nih.gov/genome/44110?genome_assembly_id=321141) | [NC_034982.1](https://www.ncbi.nlm.nih.gov/nuccore/NC_034982.1) |
| [*Ageratum conyzoides*](https://www.ncbi.nlm.nih.gov/genome/99929?genome_assembly_id=1583313) | [NC_053927.1](https://www.ncbi.nlm.nih.gov/nuccore/NC_053927.1) |
| [*Chrysanthemum boreale*](https://www.ncbi.nlm.nih.gov/genome/69078?genome_assembly_id=373149) | [NC_039757.1](https://www.ncbi.nlm.nih.gov/nuccore/NC_039757.1) |
| [*Codonopsis lanceolata*](https://www.ncbi.nlm.nih.gov/genome/70788?genome_assembly_id=385860) | [NC_037949.1](https://www.ncbi.nlm.nih.gov/nuccore/NC_037949.1) |
| [*Diplostephium hartwegii*](https://www.ncbi.nlm.nih.gov/genome/54196?genome_assembly_id=314857) | [NC_034354.1](https://www.ncbi.nlm.nih.gov/nuccore/NC_034354.1) |
| [*Helianthus annuus*](https://www.ncbi.nlm.nih.gov/genome/351?genome_assembly_id=983004) | [NC_023337.1](https://www.ncbi.nlm.nih.gov/nuccore/NC_023337.1) |
| [*Lactuca sativa*](https://www.ncbi.nlm.nih.gov/genome/352?genome_assembly_id=1684394) | [NC_042756.1](https://www.ncbi.nlm.nih.gov/nuccore/NC_042756.1) |
| [*Platycodon grandiflorus*](https://www.ncbi.nlm.nih.gov/genome/56439?genome_assembly_id=336546) | [NC_035958.1](https://www.ncbi.nlm.nih.gov/nuccore/NC_035958.1) |
| [*Daucus carota subsp. sativus*](https://www.ncbi.nlm.nih.gov/genome/860?genome_assembly_id=276486) | [NC_017855.1](https://www.ncbi.nlm.nih.gov/nuccore/NC_017855.1) |
| [*Aegiceras corniculatum*](https://www.ncbi.nlm.nih.gov/genome/85074?genome_assembly_id=1645078) | [NC_056358.1](https://www.ncbi.nlm.nih.gov/nuccore/NC_056358.1) |
| [*Camellia sinensis*](https://www.ncbi.nlm.nih.gov/genome/11029?genome_assembly_id=618300) | [NC_043914.1](https://www.ncbi.nlm.nih.gov/nuccore/NC_043914.1) |
| [*Rhododendron simsii*](https://www.ncbi.nlm.nih.gov/genome/94195?genome_assembly_id=1582937) | [NC_053763.1](https://www.ncbi.nlm.nih.gov/nuccore/NC_053763.1) |
| [*Vaccinium macrocarpon*](https://www.ncbi.nlm.nih.gov/genome/12173?genome_assembly_id=50676) | [NC_023338.1](https://www.ncbi.nlm.nih.gov/nuccore/NC_023338.1) |
| [*Cycas taitungensis*](https://www.ncbi.nlm.nih.gov/genome/10795?genome_assembly_id=39733) | [NC_010303.1](https://www.ncbi.nlm.nih.gov/nuccore/NC_010303.1) |
