# Supplementary material for: Comparative analysis of mitochondrial genomes of two alpine medicinal plants of Gentiana (Gentianaceae)
Source: PLoS One. 2023 Jan 26;18(1):e0281134. doi: 10.1371/journal.pone.0281134 (PMC9879513; doi:10.1371/journal.pone.0281134)
Supplement: S2 Table — (DOCX) [file pone.0281134.s005.docx]

**S2-1 Table** Illumina NovaSeq 6000 sequencing results.

| **Sample ID** | **Insert size (bp)** | **Raw data (Mb)** | **Clean data (Mb)** | **Clean data Q20(%)** | **Clean data Q30(%)** | **Clean data GC(%)** |
| --- | --- | --- | --- | --- | --- | --- |
| *G. crassicaulis* | 450 | 4603.1 | 4327.2 | 97.54 | 92.82 | 45.18 |
| *G. straminea* | 450 | 4818.6 | 4578.3 | 97.48 | 92.74 | 45.55 |

Insert size: insert length;

Raw data: amount of original sequencing data;

Clean data: amount of effective data;

Clean data Q20: the percentage of bases with pH value greater than 20 in the total base;

Clean data Q30: the percentage of bases with pH value greater than 30 in the total base;

Clean data GC: average GC content of effective data.

**S2-2 Table** PacBio Sequel II sequencing results.

| **Sample ID** | **Subreads number** | **Subreads bases (bp)** | **Subreads largest length (bp)** | **Subreads N50 length (bp)** | **Subreads N90 length (bp)** | **Subreads average length (bp)** |
| --- | --- | --- | --- | --- | --- | --- |
| *G. crassicaulis* | 1437127 | 12627660675 | 233271 | 9697 | 7215 | 8787 |
| *G. straminea* | 1258343 | 11503824748 | 231517 | 10079 | 7366 | 9142 |

Subreads number: the number of subreads after filtering the sample;

Subreads bases: the amount of subreads data after filtering the sample;

Subreads largest length: the largest length of subreads after filtering the sample;

Subreads N50 length: the length of subreads N50 after filtering the sample;

Subreads N90 length: the length of subreads N90 after filtering the sample;

Subreads average length: the average length of subreads after filtering the sample.
