# Supplementary material for: Comparative analysis of mitochondrial genomes of two alpine medicinal plants of Gentiana (Gentianaceae)
Source: PLoS One. 2023 Jan 26;18(1):e0281134. doi: 10.1371/journal.pone.0281134 (PMC9879513; doi:10.1371/journal.pone.0281134)
Supplement: S3 Table — (DOCX) [file pone.0281134.s006.docx]

**S3 Table** Characteristics of introns and exons in mitochondrial genes of *Gentiana crassicaulis* and *G. straminea.*

|  | Gene | Exon | Intron | Exon | Intron | Exon | Intron | Exon | Intron | Exon |
| --- | --- | --- | --- | --- | --- | --- | --- | --- | --- | --- |
|  |  | (bp) | (bp) | (bp) | (bp) | (bp) | (bp) | (bp) | (bp) | (bp) |
| *G. crassicaulis* | *nad1* | 387 | 5716 | 81 | 1142 | 192 | 25148 | 57 | 3548 | 258 |
|  | *nad2** | 153 | 1238 | 392 | ^▲^ | 161 | ^▲^ | 573 | 1672 | 188 |
|  | *nad4* | 461 | 1508 | 515 | 3389 | 423 | 2223 | 89 |  |  |
|  | *nad5** | 230 | 828 | 1216 | ^▲^ | 22 | ^▲^ | 395 | 966 | 132 |
|  | *nad7* | 143 | 960 | 69 | 1396 | 711 | 1553 | 262 |  |  |
|  | *cox1* | 726 | 949 | 867 |  |  |  |  |  |  |
|  | *cox2* | 709 | 1496 | 401 |  |  |  |  |  |  |
|  | *ccmFc* | 761 | 922 | 550 |  |  |  |  |  |  |
|  | *rps3* | 74 | 1767 | 1636 |  |  |  |  |  |  |
| *G. straminea* | *nad1** | 387 | - | 81 | 1142 | 192 | ^▲^ | 57 | 3517 | 258 |
|  | *nad2** | 153 | 1238 | 392 | ^▲^ | 161 | ^▲^ | 573 | ^▲^ | 188 |
|  | *nad4* | 461 | 1539 | 515 | 3387 | 423 | 2222 | 89 |  |  |
|  | *nad5** | 230 | 828 | 1216 | ^▲^ | 22 | ^▲^ | 395 | 966 | 132 |
|  | *nad7* | 143 | 960 | 69 | 1385 | 711 | 1553 | 262 |  |  |
|  | *cox1* | 726 | 949 | 867 |  |  |  |  |  |  |
|  | *cox2* | 709 | 1502 | 401 |  |  |  |  |  |  |
|  | *ccmFc* | 761 | 922 | 550 |  |  |  |  |  |  |
|  | *rps3* | 74 | 1785 | 1627 |  |  |  |  |  |  |

* trans-splicing gene

^▲^ trans-splicing intron
