# Supplementary material for: Comparative analysis of mitochondrial genomes of two alpine medicinal plants of Gentiana (Gentianaceae)
Source: PLoS One. 2023 Jan 26;18(1):e0281134. doi: 10.1371/journal.pone.0281134 (PMC9879513; doi:10.1371/journal.pone.0281134)
Supplement: S6 Table — (DOCX) [file pone.0281134.s009.docx]

**S6 Table** *Gentiana straminea* SNP annotation results of the reference sequence of *G. crassicaulis.*

| Sample | CDS | | | | | | | | Intergenic | Total |
| --- | --- | --- | --- | --- | --- | --- | --- | --- | --- | --- |
|  | Start_syn | Stop_syn | Start_nonsyn | Stop_nonsyn | Premature_stop | Synonymous | Nonsynonymous | Total |  |  |
| *G. straminea* | 0 | 0 | 0 | 0 | 1 | 15 | 22 | 38 | 354 | 392 |

Start_syn: start codon synonymous mutation, that is, the start codon after mutation is still the start codon; Stop_syn: stop codon synonymous mutation; Start_nonsyn: start codon nonsynonymous mutation, that is, the start codon after mutation is no longer the start codon; Stop_nonsyn: stop codon nonsynonymous mutation; Premature_stop: a nonsense mutation in which the triplet codon at the site mutates into a stop codon; Synonymous: synonymous mutation within the gene region; Nonsynonymous: nonsynonymous mutation within the gene region; Intergenic: SNP within the intergenic region.
