# Supplementary material for: Comparative analysis of mitochondrial genomes of two alpine medicinal plants of Gentiana (Gentianaceae)
Source: PLoS One. 2023 Jan 26;18(1):e0281134. doi: 10.1371/journal.pone.0281134 (PMC9879513; doi:10.1371/journal.pone.0281134)
Supplement: S7 Table — (DOCX) [file pone.0281134.s010.docx]

**S7 Table** *Gentiana straminea* InDel annotation results of the reference sequence of *G. crassicaulis.*

| Sample ID | I_gene_start | I_gene_middle | I_gene_stop | D_gene_start | D_gene_middle | D_gene_stop | Total_CDS_InDel | Intergenic | Total |
| --- | --- | --- | --- | --- | --- | --- | --- | --- | --- |
| *G. straminea* | 0 | 0 | 0 | 0 | 0 | 0 | 0 | 18 | 18 |

I_gene_start: insertion at start codon; I_gene_middle: insertion at the middle of CDS; I_gene_stop: insertion at stop codon; D_gene_start: deletion at start codon; D_gene_middle: deletion at the middle of CDS; D_gene_stop: deletion at stop codon; Total_CDS_InDel: total number of InDel located in gene region;Intergenic: InDel located in the intergenic region
