# Supplementary material for: Comparative analysis of mitochondrial genomes of two alpine medicinal plants of Gentiana (Gentianaceae)
Source: PLoS One. 2023 Jan 26;18(1):e0281134. doi: 10.1371/journal.pone.0281134 (PMC9879513; doi:10.1371/journal.pone.0281134)
Supplement: S8 Table — (DOCX) [file pone.0281134.s011.docx]

**S8 Table** Coverage statistics of collinearity comparison of *Gentiana crassicaulis* (Target) and *G. straminea* (Query).

| Target aligned (bp) | Target length (bp) | Target coverage | Query aligned (bp) | Query length (bp) | Query coverage | Number of comparis on blocks |
| --- | --- | --- | --- | --- | --- | --- |
| 224555 | 368808 | 60.89% | 224777 | 410086 | 54.81% | 209 |
