# Supplementary material for: Comparative analysis of mitochondrial genomes of two alpine medicinal plants of Gentiana (Gentianaceae)
Source: PLoS One. 2023 Jan 26;18(1):e0281134. doi: 10.1371/journal.pone.0281134 (PMC9879513; doi:10.1371/journal.pone.0281134)
Supplement: S9 Table — (DOCX) [file pone.0281134.s012.docx]

**S9 Table** Information of collinearity comparison of *Gentiana crassicaulis* (Target) and *G. straminea* (Query).

| **Target start** | **Target end** | **Query start** | **Query end** | **Target length (bp)** | **Query length (bp)** | **Direction of alignment** | **Identity** | **Score** | **Type** |
| --- | --- | --- | --- | --- | --- | --- | --- | --- | --- |
| 1718 | 2961 | 96007 | 97250 | 1244 | 1244 | + | 99.92 | 118041 | Translocation |
| 7370 | 7417 | 198889 | 198936 | 48 | 48 | + | 100 | 4620 | Translocation |
| 7470 | 7883 | 198989 | 199402 | 414 | 414 | + | 99.76 | 39424 | Translocation |
| 7906 | 8129 | 199425 | 199648 | 224 | 224 | + | 100 | 21446 | Translocation |
| 8175 | 8229 | 199694 | 199748 | 55 | 55 | + | 100 | 5302 | Translocation |
| 18583 | 19507 | 346717 | 347641 | 925 | 925 | + | 100 | 87631 | Translocation |
| 26274 | 29649 | 92161 | 95533 | 3376 | 3373 | + | 99.64 | 314528 | Translocation |
| 30119 | 30223 | 99264 | 99371 | 105 | 108 | + | 70 | 2056 | Translocation |
| 31864 | 31882 | 308930 | 308948 | 19 | 19 | + | 100 | 1783 | Translocation |
| 34136 | 34177 | 99537 | 99578 | 42 | 42 | + | 100 | 3975 | Translocation |
| 34856 | 34897 | 210033 | 210074 | 42 | 42 | + | 100 | 3993 | Translocation |
| 35353 | 35441 | 210574 | 210662 | 89 | 89 | + | 100 | 8378 | Translocation |
| 35442 | 35752 | 10708 | 11024 | 311 | 317 | + | 91.8 | 24876 | Translocation |
| 37343 | 37666 | 104097 | 104420 | 324 | 324 | + | 99.69 | 36483 | Translocation |
| 43995 | 44079 | 90928 | 91012 | 85 | 85 | + | 100 | 8050 | Translocation |
| 44642 | 45052 | 161441 | 161851 | 411 | 411 | + | 100 | 39156 | Translocation |
| 45345 | 45368 | 8531 | 8554 | 24 | 24 | + | 95.83 | 2067 | Translocation |
| 45444 | 45467 | 8631 | 8654 | 24 | 24 | + | 100 | 2247 | Translocation |
| 45480 | 46838 | 8667 | 10025 | 1359 | 1359 | + | 99.93 | 128992 | Translocation |
| 46868 | 46938 | 10055 | 10125 | 71 | 71 | + | 88.73 | 5160 | Translocation |
| 49903 | 49934 | 169801 | 169832 | 32 | 32 | + | 78.12 | 2101 | Translocation |
| 51376 | 51407 | 49242 | 49273 | 32 | 32 | + | 100 | 3038 | Translocation |
| 53328 | 58893 | 52607 | 58172 | 5566 | 5566 | + | 99.93 | 527758 | Translocation |
| 62308 | 62563 | 11695 | 11966 | 256 | 272 | + | 62.62 | 4696 | Translocation |
| 62811 | 62854 | 16450 | 16493 | 44 | 44 | + | 77.27 | 2237 | Translocation |
| 63207 | 67228 | 58173 | 62083 | 4022 | 3911 | + | 96.8 | 365682 | Translocation |
| 67281 | 68782 | 199851 | 201361 | 1502 | 1511 | + | 98.81 | 138842 | Translocation |
| 78312 | 78381 | 11293 | 11362 | 70 | 70 | + | 98.57 | 6419 | Translocation |
| 82286 | 82504 | 137227 | 137466 | 219 | 240 | + | 91.25 | 19907 | Translocation |
| 83985 | 85702 | 97257 | 98965 | 1718 | 1709 | + | 99.19 | 160058 | Translocation |
| 90317 | 92347 | 101829 | 103896 | 2031 | 2068 | + | 97.92 | 189080 | Translocation |
| 111077 | 111185 | 228789 | 228897 | 109 | 109 | + | 100 | 10441 | Translocation |
| 111460 | 111555 | 229172 | 229267 | 96 | 96 | + | 100 | 8934 | Translocation |
| 111567 | 113008 | 99633 | 101074 | 1442 | 1442 | + | 100 | 137117 | Translocation |
| 116498 | 116602 | 188601 | 188705 | 105 | 105 | + | 94.29 | 8952 | Translocation |
| 116657 | 116775 | 362423 | 362522 | 119 | 100 | + | 56.3 | 951 | Translocation |
| 118467 | 118666 | 294487 | 294686 | 200 | 200 | + | 100 | 18947 | Translocation |
| 118892 | 119091 | 294912 | 295111 | 200 | 200 | + | 100 | 18866 | Translocation |
| 124659 | 134195 | 32234 | 41828 | 9537 | 9595 | + | 98.99 | 897189 | Translocation |
| 140313 | 141352 | 236363 | 237404 | 1040 | 1042 | + | 99.71 | 96538 | Translocation |
| 142550 | 142805 | 127079 | 127334 | 256 | 256 | + | 100 | 24025 | Translocation |
| 145895 | 145987 | 188193 | 188285 | 93 | 93 | + | 100 | 8850 | Translocation |
| 159545 | 164326 | 245948 | 250719 | 4782 | 4772 | + | 99.64 | 449672 | Translocation |
| 170554 | 174728 | 86753 | 90927 | 4175 | 4175 | + | 99.78 | 394814 | Translocation |
| 181671 | 181703 | 340397 | 340429 | 33 | 33 | + | 93.94 | 2784 | Translocation |
| 182409 | 182444 | 308829 | 308863 | 36 | 35 | + | 86.11 | 1555 | Translocation |
| 183281 | 183393 | 311022 | 311121 | 113 | 100 | + | 82.3 | 6221 | Translocation |
| 184521 | 184601 | 172819 | 172920 | 81 | 102 | + | 57.84 | 1315 | Translocation |
| 186821 | 186876 | 313240 | 313297 | 56 | 58 | + | 72.88 | 1430 | Translocation |
| 188797 | 192589 | 43729 | 47521 | 3793 | 3793 | + | 100 | 360247 | Translocation |
| 193204 | 194283 | 72612 | 73691 | 1080 | 1080 | + | 99.91 | 102136 | Translocation |
| 204417 | 207648 | 314561 | 317802 | 3232 | 3242 | + | 97.83 | 303738 | Translocation |
| 207649 | 208730 | 75137 | 76217 | 1082 | 1081 | + | 99.82 | 100891 | Translocation |
| 208731 | 209157 | 13765 | 14191 | 427 | 427 | + | 100 | 40549 | Translocation |
| 221759 | 226370 | 155548 | 160153 | 4612 | 4606 | + | 99.7 | 435944 | Translocation |
| 226371 | 229092 | 77603 | 80324 | 2722 | 2722 | + | 99.89 | 258240 | Translocation |
| 243222 | 244227 | 109102 | 110106 | 1006 | 1005 | + | 98.22 | 91573 | Translocation |
| 262898 | 263025 | 334250 | 334398 | 128 | 149 | + | 85.23 | 11063 | Translocation |
| 263398 | 263644 | 123563 | 123809 | 247 | 247 | + | 100 | 23719 | Translocation |
| 263846 | 264552 | 124010 | 124716 | 707 | 707 | + | 99.86 | 66895 | Translocation |
| 277129 | 278108 | 153664 | 154643 | 980 | 980 | + | 100 | 93122 | Translocation |
| 278310 | 278733 | 154844 | 155267 | 424 | 424 | + | 100 | 40267 | Translocation |
| 278839 | 278938 | 155372 | 155471 | 100 | 100 | + | 100 | 9478 | Translocation |
| 281955 | 283366 | 180704 | 182114 | 1412 | 1411 | + | 99.72 | 132538 | Translocation |
| 283638 | 283668 | 186937 | 186967 | 31 | 31 | + | 87.1 | 2239 | Translocation |
| 308613 | 308810 | 189443 | 189597 | 198 | 155 | + | 74.75 | 12396 | Translocation |
| 319044 | 323928 | 324691 | 329594 | 4885 | 4904 | + | 99.43 | 459891 | Translocation |
| 324216 | 326881 | 276575 | 279240 | 2666 | 2666 | + | 99.77 | 251887 | Translocation |
| 327082 | 328684 | 279441 | 281043 | 1603 | 1603 | + | 99.88 | 151974 | Translocation |
| 328886 | 332255 | 281244 | 284613 | 3370 | 3370 | + | 99.97 | 319812 | Translocation |
| 333592 | 333640 | 404984 | 405036 | 49 | 53 | + | 83.02 | 1711 | Translocation |
| 334637 | 334715 | 131568 | 131640 | 79 | 73 | + | 67.09 | 1505 | Translocation |
| 335264 | 335314 | 330039 | 330092 | 51 | 54 | + | 75.93 | 2286 | Translocation |
| 335315 | 335350 | 160194 | 160229 | 36 | 36 | + | 100 | 3420 | Translocation |
| 336072 | 338296 | 131739 | 133992 | 2225 | 2254 | + | 98.23 | 208191 | Translocation |
| 338479 | 341685 | 162710 | 165916 | 3207 | 3207 | + | 99.84 | 307780 | Translocation |
| 341719 | 343383 | 165950 | 167614 | 1665 | 1665 | + | 99.88 | 157742 | Translocation |
| 344918 | 345339 | 332114 | 332535 | 422 | 422 | + | 100 | 40382 | Translocation |
| 345381 | 345407 | 227818 | 227845 | 27 | 28 | + | 60.71 | -219 | Translocation |
| 345408 | 345624 | 172017 | 172233 | 217 | 217 | + | 87.56 | 16433 | Translocation |
| 354776 | 354808 | 126938 | 126970 | 33 | 33 | + | 100 | 3093 | Translocation |
| 356958 | 357037 | 81630 | 81709 | 80 | 80 | + | 98.75 | 7345 | Translocation |
| 360038 | 360081 | 245456 | 245503 | 44 | 48 | + | 70.83 | 1749 | Translocation |
| 365052 | 365135 | 332536 | 332619 | 84 | 84 | + | 100 | 8013 | Translocation |
| 365969 | 366660 | 344212 | 344903 | 692 | 692 | + | 100 | 65960 | Translocation |
| 366661 | 366823 | 299813 | 299975 | 163 | 163 | + | 100 | 15499 | Translocation |
| 367423 | 367490 | 301484 | 301561 | 68 | 78 | + | 63.29 | 1451 | Translocation |
| 1689 | 1717 | 10026 | 10054 | 29 | 29 | - | 93.1 | 2393 | Tran+Inver |
| 3077 | 3088 | 8655 | 8666 | 12 | 12 | - | 100 | 1146 | Tran+Inver |
| 3113 | 3185 | 8555 | 8630 | 73 | 76 | - | 87.34 | 4491 | Tran+Inver |
| 3188 | 3211 | 120696 | 120719 | 24 | 24 | - | 95.83 | 2067 | Tran+Inver |
| 8275 | 10895 | 104421 | 107049 | 2621 | 2629 | - | 98.94 | 242220 | Tran+Inver |
| 11225 | 12285 | 27421 | 28483 | 1061 | 1063 | - | 98.13 | 90166 | Tran+Inver |
| 12313 | 12355 | 210672 | 210714 | 43 | 43 | - | 90.7 | 3430 | Tran+Inver |
| 14375 | 17033 | 110107 | 112765 | 2659 | 2659 | - | 99.92 | 252768 | Tran+Inver |
| 17049 | 18582 | 179025 | 180550 | 1534 | 1526 | - | 99.48 | 143951 | Tran+Inver |
| 20035 | 26273 | 352001 | 358228 | 6239 | 6228 | - | 99.71 | 591500 | Tran+Inver |
| 30463 | 31045 | 3240 | 3865 | 583 | 626 | - | 86.9 | 47269 | Tran+Inver |
| 33870 | 34101 | 161866 | 162097 | 232 | 232 | - | 100 | 21940 | Tran+Inver |
| 36288 | 37342 | 403601 | 404665 | 1055 | 1065 | - | 98.78 | 98243 | Tran+Inver |
| 40282 | 40326 | 199749 | 199793 | 45 | 45 | - | 97.78 | 4216 | Tran+Inver |
| 40381 | 40424 | 199649 | 199693 | 44 | 45 | - | 97.78 | 3174 | Tran+Inver |
| 40648 | 40669 | 199403 | 199424 | 22 | 22 | - | 100 | 2101 | Tran+Inver |
| 41087 | 41138 | 198937 | 198988 | 52 | 52 | - | 100 | 4993 | Tran+Inver |
| 42286 | 42455 | 363665 | 363834 | 170 | 170 | - | 100 | 16091 | Tran+Inver |
| 48424 | 48515 | 95837 | 95928 | 92 | 92 | - | 98.91 | 8563 | Tran+Inver |
| 51501 | 52225 | 332620 | 333344 | 725 | 725 | - | 100 | 68891 | Tran+Inver |
| 59258 | 60544 | 387045 | 388331 | 1287 | 1287 | - | 99.77 | 121843 | Tran+Inver |
| 61228 | 61264 | 80462 | 80507 | 37 | 46 | - | 53.06 | -982 | Tran+Inver |
| 61410 | 62307 | 80718 | 81615 | 898 | 898 | - | 100 | 85453 | Tran+Inver |
| 62564 | 62702 | 80325 | 80461 | 139 | 137 | - | 96.4 | 11464 | Tran+Inver |
| 62703 | 62810 | 382813 | 382920 | 108 | 108 | - | 100 | 10305 | Tran+Inver |
| 62855 | 62915 | 382708 | 382768 | 61 | 61 | - | 100 | 5803 | Tran+Inver |
| 62916 | 63064 | 127335 | 127478 | 149 | 144 | - | 92 | 10674 | Tran+Inver |
| 69173 | 72039 | 22032 | 24898 | 2867 | 2867 | - | 99.9 | 272411 | Tran+Inver |
| 72380 | 77229 | 17038 | 21882 | 4850 | 4845 | - | 99.79 | 458745 | Tran+Inver |
| 77728 | 78311 | 300769 | 301352 | 584 | 584 | - | 89.71 | 45972 | Tran+Inver |
| 82505 | 83984 | 176849 | 178328 | 1480 | 1480 | - | 99.86 | 140412 | Tran+Inver |
| 89444 | 89632 | 187013 | 187225 | 189 | 213 | - | 55.3 | 1933 | Tran+Inver |
| 92448 | 93243 | 2447 | 3239 | 796 | 793 | - | 95.48 | 64664 | Tran+Inver |
| 96439 | 96733 | 95542 | 95836 | 295 | 295 | - | 97.29 | 26481 | Tran+Inver |
| 96982 | 97453 | 322235 | 322706 | 472 | 472 | - | 99.58 | 44587 | Tran+Inver |
| 99821 | 99924 | 155268 | 155371 | 104 | 104 | - | 100 | 9869 | Tran+Inver |
| 100349 | 100548 | 154644 | 154843 | 200 | 200 | - | 100 | 19028 | Tran+Inver |
| 113109 | 113188 | 128809 | 128883 | 80 | 75 | - | 61.25 | 1783 | Tran+Inver |
| 114100 | 114806 | 207638 | 208344 | 707 | 707 | - | 99.86 | 66895 | Tran+Inver |
| 114807 | 115006 | 123810 | 124009 | 200 | 200 | - | 100 | 18992 | Tran+Inver |
| 117213 | 117393 | 65340 | 65528 | 181 | 189 | - | 94.18 | 15929 | Tran+Inver |
| 120278 | 120311 | 62218 | 62251 | 34 | 34 | - | 88.24 | 2667 | Tran+Inver |
| 122537 | 122576 | 401919 | 401958 | 40 | 40 | - | 100 | 3775 | Tran+Inver |
| 122774 | 123423 | 401064 | 401713 | 650 | 650 | - | 99.85 | 61557 | Tran+Inver |
| 123681 | 123918 | 400569 | 400806 | 238 | 238 | - | 100 | 22774 | Tran+Inver |
| 124285 | 124658 | 399829 | 400202 | 374 | 374 | - | 99.73 | 35577 | Tran+Inver |
| 134531 | 138764 | 347642 | 351869 | 4234 | 4228 | - | 99.74 | 399612 | Tran+Inver |
| 139247 | 139412 | 142656 | 142813 | 166 | 158 | - | 92.17 | 13020 | Tran+Inver |
| 139771 | 139865 | 65243 | 65339 | 95 | 97 | - | 90.72 | 6792 | Tran+Inver |
| 141453 | 142466 | 64206 | 65216 | 1014 | 1011 | - | 91.7 | 84173 | Tran+Inver |
| 145988 | 147824 | 3866 | 5662 | 1837 | 1797 | - | 96.14 | 163024 | Tran+Inver |
| 147892 | 148385 | 940 | 1436 | 494 | 497 | - | 93.76 | 41422 | Tran+Inver |
| 152229 | 153959 | 320504 | 322234 | 1731 | 1731 | - | 99.83 | 163764 | Tran+Inver |
| 153978 | 154992 | 235348 | 236362 | 1015 | 1015 | - | 99.8 | 95791 | Tran+Inver |
| 154993 | 159507 | 182115 | 186629 | 4515 | 4515 | - | 99.96 | 431742 | Tran+Inver |
| 164780 | 164826 | 227771 | 227817 | 47 | 47 | - | 68.09 | 2102 | Tran+Inver |
| 179930 | 179978 | 27051 | 27103 | 49 | 53 | - | 83.02 | 1711 | Tran+Inver |
| 185973 | 186820 | 375503 | 376350 | 848 | 848 | - | 99.88 | 80381 | Tran+Inver |
| 186877 | 188438 | 373885 | 375446 | 1562 | 1562 | - | 100 | 148739 | Tran+Inver |
| 192590 | 192751 | 384377 | 384538 | 162 | 162 | - | 96.91 | 14349 | Tran+Inver |
| 195725 | 201662 | 192982 | 198888 | 5938 | 5907 | - | 99.43 | 560552 | Tran+Inver |
| 209771 | 212805 | 173810 | 176835 | 3035 | 3026 | - | 99.28 | 285334 | Tran+Inver |
| 213522 | 214157 | 241070 | 241687 | 636 | 618 | - | 96.7 | 56437 | Tran+Inver |
| 214158 | 214183 | 172926 | 172951 | 26 | 26 | - | 96.15 | 2343 | Tran+Inver |
| 214184 | 216971 | 238205 | 241043 | 2788 | 2839 | - | 98.06 | 263027 | Tran+Inver |
| 217632 | 221116 | 138347 | 141831 | 3485 | 3485 | - | 99.97 | 330259 | Tran+Inver |
| 221117 | 221203 | 7369 | 7455 | 87 | 87 | - | 97.7 | 7858 | Tran+Inver |
| 221560 | 221624 | 362830 | 362886 | 65 | 57 | - | 80 | 2489 | Tran+Inver |
| 229175 | 230156 | 233725 | 234706 | 982 | 982 | - | 99.9 | 93542 | Tran+Inver |
| 257196 | 257655 | 318059 | 318540 | 460 | 482 | - | 95.44 | 41872 | Tran+Inver |
| 258317 | 258350 | 369812 | 369845 | 34 | 34 | - | 88.24 | 2667 | Tran+Inver |
| 258704 | 259536 | 295112 | 295944 | 833 | 833 | - | 99.88 | 78514 | Tran+Inver |
| 259738 | 259962 | 294687 | 294911 | 225 | 225 | - | 100 | 21375 | Tran+Inver |
| 260164 | 261244 | 293379 | 294486 | 1081 | 1108 | - | 97.29 | 100662 | Tran+Inver |
| 261245 | 261425 | 366535 | 366715 | 181 | 181 | - | 98.34 | 16578 | Tran+Inver |
| 261796 | 261874 | 362523 | 362647 | 79 | 125 | - | 34.06 | 348 | Tran+Inver |
| 265384 | 265643 | 101075 | 101334 | 260 | 260 | - | 100 | 24785 | Tran+Inver |
| 267193 | 267466 | 228898 | 229171 | 274 | 274 | - | 100 | 26059 | Tran+Inver |
| 267577 | 267895 | 228470 | 228788 | 319 | 319 | - | 100 | 30361 | Tran+Inver |
| 280441 | 280479 | 363974 | 364012 | 39 | 39 | - | 89.74 | 3021 | Tran+Inver |
| 280977 | 281001 | 406320 | 406344 | 25 | 25 | - | 92 | 2011 | Tran+Inver |
| 283820 | 284001 | 362648 | 362829 | 182 | 182 | - | 100 | 17282 | Tran+Inver |
| 284227 | 286801 | 359847 | 362422 | 2575 | 2576 | - | 99.57 | 243832 | Tran+Inver |
| 288029 | 288098 | 342986 | 343054 | 70 | 69 | - | 68.57 | 2026 | Tran+Inver |
| 288194 | 288228 | 50056 | 50090 | 35 | 35 | - | 77.14 | 2167 | Tran+Inver |
| 288513 | 290126 | 341131 | 342744 | 1614 | 1614 | - | 99.81 | 153351 | Tran+Inver |
| 290127 | 290207 | 67241 | 67321 | 81 | 81 | - | 98.77 | 7508 | Tran+Inver |
| 291391 | 291917 | 188916 | 189442 | 527 | 527 | - | 99.81 | 49948 | Tran+Inver |
| 291918 | 292015 | 329904 | 330008 | 98 | 105 | - | 61.9 | 1944 | Tran+Inver |
| 297748 | 297948 | 281044 | 281243 | 201 | 200 | - | 98.01 | 17697 | Tran+Inver |
| 299552 | 299751 | 279241 | 279440 | 200 | 200 | - | 100 | 18992 | Tran+Inver |
| 338358 | 338411 | 21883 | 21936 | 54 | 54 | - | 100 | 5085 | Tran+Inver |
| 355039 | 355535 | 406707 | 407203 | 497 | 497 | - | 99.2 | 46533 | Tran+Inver |
| 358790 | 359645 | 202342 | 203192 | 856 | 851 | - | 99.3 | 79383 | Tran+Inver |
| 361652 | 363641 | 205596 | 207585 | 1990 | 1990 | - | 100 | 189352 | Tran+Inver |
| 364034 | 364563 | 52070 | 52606 | 530 | 537 | - | 94.98 | 44077 | Tran+Inver |
| 366872 | 366956 | 42784 | 42874 | 85 | 91 | - | 62.64 | 2679 | Tran+Inver |
| 367109 | 367350 | 237966 | 238204 | 242 | 239 | - | 95.04 | 20290 | Tran+Inver |
| 367563 | 367678 | 237405 | 237520 | 116 | 116 | - | 81.9 | 7100 | Tran+Inver |
| 367806 | 368287 | 24899 | 25380 | 482 | 482 | - | 99.59 | 45486 | Tran+Inver |
| 189 | 1688 | 124938 | 126437 | 1500 | 1500 | - | 99.93 | 142253 | Inversion |
| 32348 | 33438 | 134314 | 135404 | 1091 | 1091 | - | 99.91 | 103776 | Inversion |
| 60545 | 61202 | 149370 | 150027 | 658 | 658 | - | 99.85 | 62247 | Inversion |
| 61265 | 61389 | 188754 | 188878 | 125 | 125 | - | 100 | 11852 | Inversion |
| 78454 | 81834 | 189598 | 192981 | 3381 | 3384 | - | 99.85 | 319247 | Inversion |
| 93750 | 94840 | 217942 | 219032 | 1091 | 1091 | - | 99.91 | 103776 | Inversion |
| 267896 | 268487 | 69931 | 70515 | 592 | 585 | - | 98.82 | 55424 | Inversion |
| 297949 | 298157 | 91013 | 91221 | 209 | 209 | - | 100 | 19775 | Inversion |
| 101512 | 110757 | 250720 | 259964 | 9246 | 9245 | + | 99.72 | 873999 | Colinearity |
| 148386 | 152228 | 272642 | 276485 | 3843 | 3844 | + | 99.87 | 364503 | Colinearity |
| 170465 | 170553 | 276486 | 276574 | 89 | 89 | + | 100 | 8423 | Colinearity |
| 230231 | 236365 | 302732 | 308828 | 6135 | 6097 | + | 97.85 | 570893 | Colinearity |
| 236366 | 238438 | 308949 | 311021 | 2073 | 2073 | + | 100 | 197274 | Colinearity |
| 244438 | 253973 | 390232 | 399828 | 9536 | 9597 | + | 98.99 | 896341 | Colinearity |
| 254350 | 254715 | 400203 | 400568 | 366 | 366 | + | 100 | 34656 | Colinearity |
| 254956 | 255212 | 400807 | 401063 | 257 | 257 | + | 100 | 24476 | Colinearity |
| 255847 | 256051 | 401714 | 401918 | 205 | 205 | + | 98.05 | 18674 | Colinearity |
| 256092 | 257195 | 401959 | 403062 | 1104 | 1104 | + | 99.73 | 104502 | Colinearity |
| 264582 | 265383 | 6499 | 7300 | 802 | 802 | + | 98.38 | 73201 | Colinearity |
| 338320 | 338340 | 99612 | 99632 | 21 | 21 | + | 80.95 | 1412 | Colinearity |
| 341686 | 341718 | 101757 | 101789 | 33 | 33 | + | 96.97 | 3052 | Colinearity |
| 345760 | 353562 | 112854 | 120695 | 7803 | 7842 | + | 98.07 | 728936 | Colinearity |
