# Supplementary material for: Comparative analysis of mitochondrial genomes of two alpine medicinal plants of Gentiana (Gentianaceae)
Source: PLoS One. 2023 Jan 26;18(1):e0281134. doi: 10.1371/journal.pone.0281134 (PMC9879513; doi:10.1371/journal.pone.0281134)
Supplement: S11 Table — (DOCX) [file pone.0281134.s014.docx]

**S11-1 Table** Information exchange and transfer between chloroplasts and mitochondria in *Gentiana crassicaulis.*

| No. | Chloroplast genome of *G. crassicaulis* | | Chloroplast genes carried | Mitochondrial genome of *G. crassicaulis* | | Identity | Length |
| --- | --- | --- | --- | --- | --- | --- | --- |
|  | Start | End |  | Start | End |  |  |
| 1 | 8874 | 9630 | *atpA* (partial) | 123351 | 122603 | 0.98 | 757 |
| 2 | 8874 | 9630 | *atpA* (partial) | 255285 | 256025 | 0.91 | 768 |
| 3 | 30961 | 31956 | *psbD* (partial) | 166377 | 165387 | 0.98 | 998 |
| 4 | 30961 | 31956 | *psbD* (partial) | 306594 | 307584 | 0.98 | 998 |
| 5 | 30961 | 31956 | *psbD* (partial) | 320039 | 319044 | 1 | 996 |
| 6 | 63786 | 63926 | ***trnW-CCA*** | 148700 | 148561 | 0.92 | 141 |
| 7 | 70540 | 70805 | *psbB* (partial) | 63323 | 63058 | 0.99 | 266 |
| 8 | 82312 | 83393 | *rpl2* (partial)-*rpl23*-*trnI-CAU* | 207649 | 208730 | 1 | 1082 |
| 9 | 93827 | 97148 | *rps7*-*rps12*-***trnV-GAC***-*rrn16* (partial) | 119924 | 116603 | 0.99 | 3322 |
| 10 | 93827 | 97148 | *rps7*-*rps12*-***trnV-GAC***-*rrn16* (partial) | 258704 | 262047 | 0.99 | 3344 |
| 11 | 97077 | 99593 | *rrn16*-*trnI-GAU* (partial) | 201654 | 204170 | 0.99 | 2517 |
| 12 | 100048 | 106415 | *trnA-UGC* (partial)-*rrn23*-*rrn4.5*-*rrn5*-*trnR-ACG*-*trnN-GUU*-*Ψycf1* | 155218 | 161585 | 1 | 6368 |
| 13 | 104473 | 106415 | *trnN-GUU*-*Ψycf1* | 313300 | 315242 | 0.99 | 1943 |
| 14 | 104473 | 106415 | *trnN-GUU*-*Ψycf1* | 313328 | 311386 | 1 | 1943 |
| 15 | 123303 | 125428 | *ycf1* (partial)-*trnN-GUU* | 311203 | 313328 | 0.99 | 2126 |
| 16 | 123303 | 125428 | *ycf1* (partial)-*trnN-GUU* | 315425 | 313300 | 0.99 | 2126 |
| 17 | 123303 | 129853 | *ycf1* (partial)-*trnN-GUU*-*trnR-ACG*-*rrn5*-*rrn4.5*-*rrn23*-*trnA-UGC* (partial) | 161768 | 155218 | 0.99 | 6551 |
| 18 | 130308 | 132824 | *trnI-GAU* (partial)-*rrn16* (partial) | 204170 | 201654 | 0.99 | 2517 |
| 19 | 132753 | 136074 | *rrn16* (partial)-***trnV-GAC***-*rps12*-*rps7* | 116603 | 119924 | 0.99 | 3322 |
| 20 | 132753 | 136074 | *rrn16* (partial)-***trnV-GAC***-*rps12*-*rps7* | 262047 | 258704 | 0.99 | 3344 |
| 21 | 146508 | 147589 | ***trnI-CAU***-*rpl23*-*rpl2* (partial) | 208730 | 207649 | 1 | 1082 |

**S11-2 Table** Information exchange and transfer between chloroplasts and mitochondria in *G. straminea.*

| No. | Chloroplast genome of *G. straminea* | | Chloroplast genes carried | Mitochondrial genome of *G. straminea* | | Identity | Length |
| --- | --- | --- | --- | --- | --- | --- | --- |
|  | Start | End |  | Start | End |  |  |
| 1 | 9003 | 9757 | *atpA* | 401138 | 401892 | 1 | 755 |
| 2 | 17271 | 19319 | *rpoC2* (partial)-*rpoC1* (partial) | 367907 | 369955 | 0.99 | 2049 |
| 3 | 17271 | 19319 | *rpoC2* (partial)-*rpoC1* (partial) | 64156 | 62108 | 0.99 | 2049 |
| 4 | 31112 | 34105 | *psbD*-*psbC* | 325686 | 322693 | 0.99 | 2994 |
| 5 | 34530 | 34839 | *psbZ* | 169455 | 169146 | 1 | 310 |
| 6 | 50722 | 56897 | *atpE* (partial)-*atpB*-*rbcL*-*accD* | 290772 | 284597 | 0.99 | 6176 |
| 7 | 63896 | 64035 | ***trnW-CCA*** | 272957 | 272817 | 0.91 | 141 |
| 8 | 63896 | 64035 | ***trnW-CCA*** | 83135 | 82995 | 0.91 | 141 |
| 9 | 63927 | 64035 | ***trnW-CCA*** | 656 | 764 | 0.94 | 109 |
| 10 | 64496 | 64643 | None | 511 | 658 | 1 | 148 |
| 11 | 80960 | 84829 | *rpl22* (partial)-*rps19*-*rpl2*-*rpl23*-***trnI-CAU***-*ycf2* (partial) | 263137 | 267108 | 0.95 | 3981 |
| 12 | 80960 | 84830 | *rpl22* (partial)-*rps19*-*rpl2*-*rpl23*-***trnI-CAU***-*ycf2* (partial) | 73688 | 77557 | 0.99 | 3872 |
| 13 | 90802 | 96488 | *trnL-CAA*-*ndhB*-*rps7*-*rps12* | 299065 | 293379 | 0.99 | 5687 |
| 14 | 96455 | 96663 | *trnV-GAC* (partial) | 366749 | 366541 | 1 | 209 |
| 15 | 96455 | 96663 | *trnV-GAC* (partial) | 65314 | 65522 | 1 | 209 |
| 16 | 100189 | 104463 | *trnA-UGC* (partial)-*rrn23*-*rrn4.5*-*rrn5* | 186404 | 182129 | 0.99 | 4276 |
| 17 | 104533 | 106573 | *trnR-ACG*-***trnN-GUU***-*ψycf1* | 245948 | 247988 | 1 | 2041 |
| 18 | 123476 | 125699 | *ycf1* (partial)-***trnN-GUU***-*trnR-ACG* | 248171 | 245948 | 0.99 | 2224 |
| 19 | 125769 | 130043 | *rrn5*-*rrn4.5*-*rrn23*-*trnA-UGC* (partial) | 182129 | 186404 | 0.99 | 4276 |
| 20 | 133569 | 133777 | *trnV-GAC* (partial) | 366541 | 366749 | 1 | 209 |
| 21 | 133569 | 133777 | *trnV-GAC* (partial) | 65522 | 65314 | 1 | 209 |
| 22 | 133744 | 139430 | *rps12*-*rps7*-*ndhB*-*trnL-CAA* | 293379 | 299065 | 0.99 | 5687 |
| 23 | 145402 | 148991 | *ycf2* (partial)-***trnI-CAU***-*rpl23*-*rpl2*-*rps19* | 77557 | 73969 | 0.99 | 3591 |
| 24 | 145403 | 148991 | *ycf2* (partial)-***trnI-CAU***-*rpl23*-*rpl2*-*rps19* | 267108 | 263418 | 0.95 | 3700 |
