# Supplementary material for: Comparative analysis of mitochondrial genomes of two alpine medicinal plants of Gentiana (Gentianaceae)
Source: PLoS One. 2023 Jan 26;18(1):e0281134. doi: 10.1371/journal.pone.0281134 (PMC9879513; doi:10.1371/journal.pone.0281134)
Supplement: S12 Table — (DOCX) [file pone.0281134.s015.docx]

**S12 Table** Pan genes of 6 Gentianales plants.

| **Gene ID** | ***Gentiana crassicaulis*** | ***Gentiana straminea*** | ***Asclepias syriaca* (NC022796)** | ***Rhazya stricta* (NC024293)** | ***Cynanchum auriculatum* (NC041494)** | ***Scyphiphora hydrophyllacea* (NC057654)** |
| --- | --- | --- | --- | --- | --- | --- |
| GU08_p39(NC024293) ^*^ | 98.34 | 98.34 | 95.96 | 100 | 100 | 98.81 |
| GU08_p35(NC024293) ^*^ | 94.32 | 94.32 | 78.11 | 100 | 98.02 | 97.1 |
| V566_p15(NC022796) | 87.15 | 87.15 | 100 | 91.21 | 0 | 69.56 |
| rps3(*G. crassicaulis*) ^*^ | 100 | 98.76 | 89.91 | 92.54 | 92.01 | 91.31 |
| cox1(*G. crassicaulis*) | 100 | 100 | 95.81 | 97.53 | 0 | 96.77 |
| atp1(*G. crassicaulis*) ^*^ | 100 | 99.8 | 96.45 | 97.64 | 96.27 | 98.62 |
| nad4(*G. crassicaulis*) | 100 | 100 | 92.32 | 96.57 | 0 | 95.76 |
| nad2(*G. crassicaulis*) ^*^ | 100 | 99.8 | 93.24 | 98.16 | 97.54 | 97.95 |
| HXU36_mgp17(NC057654) ^*^ | 90.37 | 90.37 | 87.82 | 93.33 | 91.72 | 100 |
| *atp4*(*G. straminea*) | 100 | 100 | 86.02 | 85.35 | 0 | 85.64 |
| HXU36_mgp38(NC057654) | 0 | 0 | 0 | 0 | 89.2 | 100 |
| nad7(*G. crassicaulis*) ^*^ | 100 | 100 | 93.91 | 97.21 | 96.95 | 96.44 |
| V566_p26(NC022796) ^*^ | 94.9 | 94.87 | 100 | 96.69 | 97.2 | 96.69 |
| GU08_p33(NC024293) ^*^ | 94.03 | 93.75 | 93.43 | 100 | 97.46 | 95.47 |
| atp6(*G. crassicaulis*) | 100 | 0 | 0 | 93.01 | 0 | 0 |
| cox2(*G. crassicaulis*) ^*^ | 100 | 100 | 87.94 | 91.76 | 91.05 | 91.47 |
| GU08_p31(NC024293) | 0 | 0 | 0 | 100 | 92.01 | 94.8 |
| V566_p08(NC022796) ^*^ | 94.14 | 94.14 | 100 | 94.44 | 94.77 | 93.54 |
| V566_p40(NC022796)^▲^ | 0 | 0 | 100 | 0 | 0 | 0 |
| GU08_p49(NC024293) | 95.47 | 95.06 | 77.55 | 100 | 0 | 92.22 |
| cox3(*G. crassicaulis*) ^*^ | 100 | 100 | 93.21 | 96.6 | 96.6 | 96.6 |
| ccmC(*G. crassicaulis*) ^*^ | 100 | 100 | 84.45 | 93.6 | 91.6 | 93.59 |
| *nad6(G. straminea*) ^*^ | 99.11 | 100 | 93.66 | 97.07 | 97.07 | 94.52 |
| rps1(*G. crassicaulis*) | 100 | 100 | 84.97 | 87.88 | 0 | 0 |
| ccmB(*G. crassicaulis*) | 100 | 100 | 84.47 | 97.57 | 0 | 97.09 |
| HXU36_mgp04(NC057654) | 0 | 0 | 0 | 0 | 84.18 | 100 |
| nad9(*G. crassicaulis*) ^*^ | 100 | 98.95 | 95.26 | 96.84 | 96.84 | 96.84 |
| rpl5(*G. crassicaulis*) | 100 | 100 | 89.2 | 94.02 | 0 | 93.48 |
| HXU36_mgp14(NC057654) | 94.15 | 94.15 | 0 | 96.49 | 0 | 100 |
| V566_p33(NC022796) | 0 | 0 | 100 | 95.68 | 0 | 95.06 |
| E6S41_mgp23(NC041494) ^*^ | 89.31 | 89.31 | 97.47 | 89.81 | 100 | 86.16 |
| GU08_p20(NC024293) ^*^ | 90.48 | 90.48 | 90.91 | 100 | 98.48 | 96.95 |
| V566_p02(NC022796) | 0 | 0 | 100 | 91.89 | 97.97 | 89.86 |
| GU08_p16(NC024293) | 0 | 0 | 92.59 | 100 | 0 | 93.69 |
| rps12(*G. crassicaulis*) | 100 | 100 | 95.2 | 96.8 | 0 | 96 |
| nad3(*G. crassicaulis*) | 100 | 100 | 87.29 | 91.53 | 0 | 89.83 |
| rps13(*G. crassicaulis*) ^*^ | 100 | 100 | 91.38 | 95.69 | 95.69 | 97.41 |
| E6S41_mgp05(NC041494) | 0 | 0 | 90.83 | 0 | 100 | 0 |
| nad4L(*G. crassicaulis*) | 100 | 100 | 86 | 97 | 0 | 96 |
| GU08_p26(NC024293) | 0 | 0 | 94.57 | 100 | 97.87 | 0 |
| GU08_p29(NC024293)^▲^ | 0 | 0 | 0 | 100 | 0 | 0 |
| GU08_p37(NC024293) | 89.19 | 89.19 | 89.19 | 100 | 0 | 96 |

*Core gene

^▲^Specific genes
